# Unsupervised Mixture Models on the Edge for Smart Energy Consumption Segmentation with Feature Saliency

**DOI:** 10.3390/s23198296

**Published:** 2023-10-07

**Authors:** Hussein Al-Bazzaz, Muhammad Azam, Manar Amayri, Nizar Bouguila

**Affiliations:** Concordia’s Institute for Information Systems Engineering (CIISE), Concordia University, Montreal, QC H3G 1M8, Canadamanar.amayri@concordia.ca (M.A.); nizar.bouguila@concordia.ca (N.B.)

**Keywords:** probabilistic modelling, energy analytics, bounded mixture models, asymmetric generalized Gaussian distribution, feature selection

## Abstract

Smart meter datasets have recently transitioned from monthly intervals to one-second granularity, yielding invaluable insights for diverse metering functions. Clustering analysis, a fundamental data mining technique, is extensively applied to discern unique energy consumption patterns. However, the advent of high-resolution smart meter data brings forth formidable challenges, including non-Gaussian data distributions, unknown cluster counts, and varying feature importance within high-dimensional spaces. This article introduces an innovative learning framework integrating the expectation-maximization algorithm with the minimum message length criterion. This unified approach enables concurrent feature and model selection, finely tuned for the proposed bounded asymmetric generalized Gaussian mixture model with feature saliency. Our experiments aim to replicate an efficient smart meter data analysis scenario by incorporating three distinct feature extraction methods. We rigorously validate the clustering efficacy of our proposed algorithm against several state-of-the-art approaches, employing diverse performance metrics across synthetic and real smart meter datasets. The clusters that we identify effectively highlight variations in residential energy consumption, furnishing utility companies with actionable insights for targeted demand reduction efforts. Moreover, we demonstrate our method’s robustness and real-world applicability by harnessing Concordia’s High-Performance Computing infrastructure. This facilitates efficient energy pattern characterization, particularly within smart meter environments involving edge cloud computing. Finally, we emphasize that our proposed mixture model outperforms three other models in this paper’s comparative study. We achieve superior performance compared to the non-bounded variant of the proposed mixture model by an average percentage improvement of 7.828%.

## 1. Introduction

The predictive power of machine learning holds the key to deciphering intricate patterns and driving efficient solutions for a sustainable future, particularly in the realm of smart meter data modelling and utility program improvement. This predictive capability has already played a crucial role in ensuring global food supplies, a once seemingly insurmountable challenge. Scientists have been instrumental in harnessing this power for the benefit of society. As we embark on this new era, machine learning is poised to further our understanding of the world, optimize resource utilization, and reduce our environmental impact, ultimately promoting prosperity and sustainability. In this pursuit, our focus is on the intricacies of smart meter data modelling and its application in enhancing utility programs, such as energy efficiency and demand response. The implementation of Advanced Metering Infrastructure (AMI) across Europe stands as a notable catalyst behind the surpassing of energy efficiency targets outlined in the EU’s 20-20-20 energy policy. Building on the triumphs in Europe, smart meter deployments have transcended borders, becoming a global phenomenon in nations striving to modernize their electricity grids. Consequently, these groundbreaking advancements in energy metering technologies have birthed a trove of high-quality, consistently sampled electrical power consumption datasets. This surge in data dimensions underscores the compelling necessity for meticulous feature selection within the domain of machine learning models. This ensures the prioritization of the most enlightening attributes while simultaneously mitigating noise and curtailing computational expenditures. Within the machine learning context, “features” denote the distinct measurable properties or intrinsic characteristics of data that serve as the essential input for predictive models. In the scope of this paper, when we allude to “features”, we specifically refer to the statistical metrics derived from time-series data or the readings gleaned from smart meters pertaining to a particular energy consumer. Our work delves into the challenges and potential of this domain, introducing methodologies that not only improve the predictive accuracy but also enhance transparency and interpretability. Our goal is to ensure that every stakeholder, from scientists to policymakers, can fully utilize the potential of these advancements for a brighter and more sustainable future.

The challenge of smart meter data modelling using clustering techniques is pivotal in advancing utility programs geared towards achieving energy sustainability and fostering a better future. In this context, the integrated IoT architecture for smart metering proposed by the research in [1] provides valuable insights into the technological foundations of modern smart metering systems. Effectively harnessing high-frequency smart meter data to understand consumer energy consumption behaviour presents a significant opportunity. Research papers, exemplified by [2], have delved into the segmentation of household energy consumption using hourly data, enabling the identification of intricate consumption patterns. Likewise, the work in [3] revolves around the analysis and clustering of residential customers’ energy behavioural demand using smart meter data, facilitating the recognition of distinct consumption behaviours [3,4,5,6,7]. These modelling solutions offer substantial benefits by providing utility programs with tailored insights. They empower utilities to develop strategies for energy efficiency and demand response that are intricately aligned with consumer behaviour. Ultimately, this not only enhances energy sustainability but also contributes to the creation of a more environmentally responsible and prosperous future.

**Figure 1:**
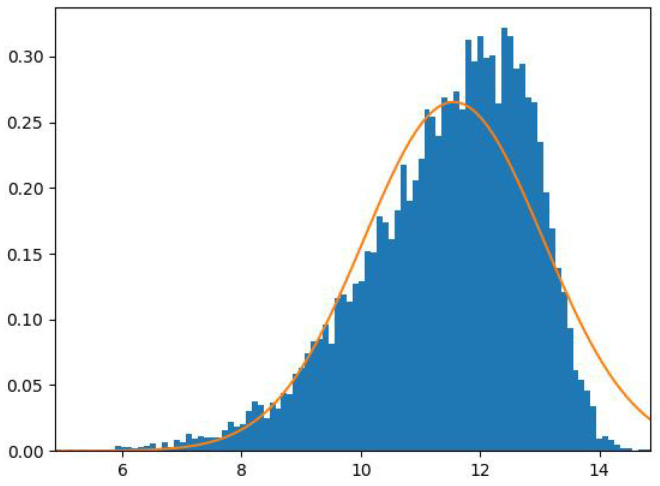
The Gaussian distribution symmetry problem.

Moreover, the richer and more granular data may lead to more complex and diverse consumption patterns, necessitating the use of flexible distributions in statistical models to capture the nuances in class data distributions effectively [8,9,10]. DR is an incentive program that allows utility companies to save money on unnecessary investments and lower emissions of greenhouse gases (GHG) [8,9,10]. DR induces households to reduce their energy consumption levels at high wholesale market prices or when system reliability is jeopardized. EE programs aim to reduce the power demand of households while maintaining their consumption habits [8,11,12,13]. Traditional machine learning exploratory analysis tools, such as unsupervised learning techniques, transform smart meter information into valuable information participating in customer clustering [8]. Clustering is a statistical data analysis technique that can uncover or infer intrinsic properties and cluster the data into several components according to the observations’ similarities [8]. As a soft clustering approach, the Gaussian mixture’s reliability and minimal impact on computational capabilities have made it a good candidate for modelling smart meter data [8,14,15,16]. The Gaussian distribution does not fit data well within a mixture model if the data have an asymmetric distribution, as demonstrated in Figure 1. The estimation of data-bounded support regions using Gaussian mixture models has been a notable avenue of research, with advancements in vector quantization techniques [17,18,19,20,21]. The deployment of AMI has introduced high dimensionality in modern energy consumption datasets [4]. Patterns are easily distinguished within observations represented with features of high entropy. Feature selection has several advantages: it is well established to improve the performance of model-based classification [22], and it helps to develop interpretable models that are reduced in complexity within applications across several disciplines [23]. The search for the optimal number of clusters and the optimal set of features is an interrelated optimization problem [23]. However, searching for the optimal set of features is challenging in an unsupervised setting because there is no clear criterion for the optimization process, since the number of clusters is unknown [23]. Historically, to find the optimal number of features, an exhaustive search is done through the space of all feature subsets [24,25,26]. Additionally, non-exhaustive search techniques do not guarantee finding the optimal feature subset. Therefore, an efficient solution was proposed within an unsupervised setting [23]; the optimal feature subset search is converted into an estimation problem parallel to the learning of mixture models, where a vector of feature weights is estimated using the expectation-maximization (EM) algorithm [23].

In our experimental analysis, our proposed method outperforms the asymmetric generalized Gaussian mixture model-based feature selection (FSAGGMM), the bounded asymmetric generalized Gaussian mixture model (BAGGMM), and the asymmetric generalized Gaussian mixture model (AGGMM) according to several performance evaluation metrics. Additionally, our proposed mixture model has been implemented using Concordia University’s High-Performance Computing (HPC) Facility: Speed [27].

The current energy consumer segmentation approach distinguishes itself from previous works by effectively modelling different representations of smart meter data, taking into account the class data bounds, inferring the true number of consumer clusters, and finding the optimal set of features in a single optimization process. The rest of the paper is organized as follows: in Section 2, we inform the reader about all the prior works within the context of this paper. in Section 3, we describe the proposed feature selection model based on the bounded asymmetric generalized Gaussian mixture model (FSBAGGMM). Section 4 explains how the mixture model’s parameters are estimated and how the MML’s objective function is derived for our specific case. Section 5 exhibits the experimental results in the context of household energy consumption segmentation by comparing the performance of our proposed algorithm against several state-of-the-art clustering algorithms. Finally, we discuss and conclude our research in Section 6 and Section 7, respectively.

## 2. Prior Works

Numerous applications leverage energy consumption data, benefiting from the increased feasibility and reliability facilitated by smart meters. Non-intrusive load monitoring (NILM) has enhanced heating, ventilation, and air conditioning (HVAC) fault detection through smart meter readings, eliminating the need for additional sensors [28]. Smart meter data serve as valuable input for load forecasting and energy efficiency recommendations [29]. Customer-oriented solutions, such as user-friendly web portals for bill understanding, have also been proposed [30]. Additionally, energy consumption data inform predictive models and offer consumption insights, further contributing to energy efficiency [29,31,32]. Previous research has addressed key aspects of smart meter data analytics. The research in [33] focused on smart-meter-driven segmentation, while the research in [34] introduced layer-wise relevance propagation for smart grid stability prediction. The research in [35] optimized deep models for improved smart grid stability prediction. Additionally, the research in [36] explored customer segmentation based on smart meter data analytics. These studies form the foundation for our research, covering various aspects of smart meter data analysis and its applications.

Clustering has proven helpful to find energy consumption patterns in low- and high-voltage customers [37,38]. Additionally, demand management programs have successfully utilized clustering in order to select suitable candidate energy consumers [39,40,41]. Thus, several approaches have been employed for the segmentation of energy users, such as Euclidean distance-based clustering [31,38] and multi-resolution clustering in the spectral domain [42]. Similarly, several clustering methods, such as hierarchical clustering, K-means, fuzzy K-means, and self-organizing maps (SOM), have been used to cluster consumers with similar energy consumption patterns in [37]. SOM was tested for its capability to classify consumption profiles in [43]. Clustering has also proven useful to enhance energy consumption prediction using a two-layer feed-forward artificial neural network [10]. The Gaussian mixture model, optimized by the EM algorithm, was utilized in [32,44] as a non-distance-based consumer segmentation tool. Other finite mixture models have also been used within the context of the same application [45].

In order to model smart meter data in different representations, several limitations imposed by the Gaussian mixture model must be overcome. Several distributions have been used as a base distribution of mixture models to overcome the shape rigidity of the Gaussian distribution, such as the Student’s-t distribution [46,47,48] and the generalized Gaussian distribution (GGD) [49,50,51]. Compared to the Gaussian distribution, the Student’s-t distribution has an additional parameter (ν) called the degree of freedom that allows the distribution to generalize to different probability distributions. The Student’s-t distribution is identical to the Cauchy distribution when (ν=1) and approaches the Gaussian distribution as (ν) approaches infinity. As for the GGD, the additional parameter per component (λ) is called the shape parameter; it controls the tails of the distribution, making it far more flexible to different types of data and less vulnerable to outliers [52,53,54]. In more recent studies, the asymmetric generalized Gaussian distribution (AGGD) was used as a base distribution for mixture models [55,56]. The AGGD can generalize to a large class of distributions, such as the impulsive, the Laplacian, the Gaussian, and the uniform distributions, in addition to the ability to fit asymmetric data [57]. Additionally, and in order for mixture components to fit better to real-life data, the bounded support concept was adopted in several finite mixture models [17,20,58].

Several feature extraction methods have been utilized to process high-dimensional data in electrical load observations and convert them into a new set of reduced feature spaces. In [59], a scalable algorithm for data processing has been proposed for a dataset collected from 10,000 Australian homes over a year. Dimensionality reduction is accomplished by employing a sparse representation technique in [60]. An encoding system has given representations for energy consumers with a pre-processed dictionary in [2]. The discovery of prominent energy consumption time windows is crucial for feature extraction and, therefore, in modelling the typical consumer’s behaviour. Through a thorough analysis of several smart meter trials, researchers have been able to identify four time periods where the most extensive distribution of peak demand occurs within smart meter datasets [32]. The energy consumption data within the specified time periods were used to calculate seven weakly correlated features. Projection methods such as principal component analysis (PCA) were also used to concisely represent a consumer’s load curve [37].

In the context of the energy consumption segmentation application, a feature selection approach based on genetic algorithms has been utilized effectively in [31] to reduce the high dimensionality of smart meter data and improve the clustering performance of k-means. In general, several exhaustive search methods are conducted to perform feature selection, such as sequential forward search, backward search, floating search, beam search, bidirectional search, and genetic search [24,25,26,61]. However, more recently, several studies have approached the problem of finding the optimal set of features as an optimization problem within the context of mixture-based clustering in several real-life applications [56,62], thus achieving feature selection with minimal computation expenses.

Various methods have been employed to determine the optimal number of energy consumer clusters. Diverse clustering evaluation metrics and scenarios have been utilized, with the best scenario dictating the optimal number of consumption profiles [63,64]. Additionally, an entropy-based evaluation index was applied to time series data for cluster optimization [31]. Probabilistic model selection methods, such as the Bayesian Information Criterion (BIC) and the Akaike Information Criterion (AIC), were used in different studies to select the optimal cluster count [32,65]. It is worth noting that the AIC tends to favour more complex models, particularly with smaller training datasets, while the BIC leans toward simpler models. Another superior approach is the Minimum Message Length (MML) criterion, known for its excellence over BIC and AIC [66,67,68]. MML, combined with the feature-weighting mixture model [23], simultaneously performs model and feature selection, avoiding exhaustive searches. This paper builds on prior research that has evolved mixture models to become increasingly flexible and assumption-light, aiming to better capture real-world data complexities. Our proposed model leverages this accumulated knowledge to introduce a more flexible approach.

## 3. The Unsupervised BAGGMM-Based Feature Selection Model

Mixture models are a powerful approach to model incomplete data. The observations in this paper are represented as a set of vectors $X=\{\vec{X_{1}},\vec{X_{2}},\vec{X_{3}},\ldots ,\vec{X_{N}}\}$, $\vec{X_{i}}\in R^{D}$, i∈{1,2,3,…,N}. We aim to model data in X using a mixture model with *M* components where M≥1. It is possible to state that the *D*-dimensional random variable ${\vec{X}}_{i}=\left(X_{i1},X_{i2},\ldots ,X_{iD}\right)$ is sampled from a *M* component mixture model if its probability density function can be written as follows:

(1) $$p\left({\vec{X}}_{i}|\Theta \right)=\sum_{k=1}^{M}p\left({\vec{X}}_{i}|\theta_{k}\right)p_{k}$$

where Θ represents the set of parameters of all the M-component mixture models. The term $p_{k}$ represents the mixing proportion of the component *k*; by definition, $p_{k}$ is positive and $\sum_{k=1}^{M}p_{k}=1$. The likelihood function gives the joint distribution for all the observations:

(2) $$p\left(X|\Theta \right)=\prod_{i=1}^{N}\sum_{k=1}^{M}p\left({\vec{X}}_{i}|\theta_{k}\right)p_{k}$$

In order to define the complete data likelihood, an *M*-dimensional vector of unobserved variables is defined, and it is denoted by $\vec{Z_{i}}$. For each observation *i*, the unobserved binary vector is assigned with 0 s, except at the *k*’th position, where the cluster is responsible primarily. The complete data likelihood is defined as follows:

(3) $$p\left(X,Z|\Theta \right)=\prod_{i=1}^{N}\prod_{k=1}^{M}{\left(p\left(\vec{X_{i}}|\theta_{k}\right)p_{k}\right)}^{Z_{ik}}$$

where $Z=\{\vec{Z_{1}},\ldots ,\vec{Z_{N}}\}$. The features in Equation (2) are considered to be of equal importance. However, in the context of a real application, the estimation of the feature weights is an effective approach to better model data [37,38]. The integration of the feature selection approach within the mixture model involves considering that the irrelevant features are modelled with a background Gaussian distribution as in [23]. In this paper, feature weights are estimated for all the mixture components. Therefore, the background Gaussian distribution has a single set of parameters $\vec{\beta }=\left\{\vec{\eta },\vec{\delta }\right\}$, where $\vec{\eta }$ represents the vector of means for all the data dimensions and $\vec{\delta }$ represents the standard deviation vector. Thus, we are proposing to rewrite Equation (2) to adopt feature relevancy as follows:

(4) $$p\left(\vec{X_{i}}|\Theta ,\vec{\beta },\vec{\varphi }\right)=\sum_{k=1}^{M}p_{j}\prod_{d=1}^{D}p{\left(X_{id}|\theta_{kd}\right)}^{\varphi_{d}}p{\left(X_{id}|\beta_{d}\right)}^{1-\varphi_{d}}$$

where $\vec{\beta }=\left\{\left(\eta_{1},\delta_{1}\right),\cdots ,\left(\eta_{D},\delta_{D}\right)\right\}$. The unobserved binary vector $\vec{\varphi }=\left(\varphi_{1},\cdots ,\varphi_{D}\right)$ indicates the relevancy of each feature. By assuming that the elements within vector $\vec{\varphi }$ are mutually exclusive and independent of the component label *Z*, we have

(5) $$p\left(\vec{X_{i}},\vec{\varphi }\right)=p\left(\vec{X_{i}}|\vec{\varphi }\right)p\left(\vec{\varphi }\right)=\sum_{k=1}^{M}p_{k}\prod_{d=1}^{D}{\left(\omega_{d}p\left(X_{id}|\theta_{kd}\right)\right)}^{\varphi_{d}}\times {\left(\left(1-\omega_{d}\right)p\left(X_{id}|\beta_{d}\right)\right)}^{1-\varphi_{d}}$$

After the marginalization over φ, the obtained mixture model is formalized as follows:

(6) $$p\left(\vec{X_{i}}|\Theta_{M}\right)=\sum_{k=1}^{M}p_{k}\prod_{d=1}^{D}\left[\omega_{d}p\left(X_{id}|\theta_{kd}\right)+\left(1-\omega_{d}\right)p\left(X_{id}|\beta_{d}\right)\right]$$

where $\Theta_{M}$ = [$\Theta ,\vec{\omega },\vec{\beta }$] is the complete set of parameters that define the proposed mixture model. The vector $\vec{\omega }=\left(\omega_{1},\ldots ,\omega_{D}\right)$ quantifies the feature importance with a set of weights where $\omega_{d}=p\left(\varphi_{d}=1\right)$. Thus, Equation (6) represents the probability density function that is assumed to generate the data. The foreground distribution or the mixture base distribution $p(X_{id}|\theta_{kd})$ models the relevant attributes of each latent class in the data. Several distributions have been proposed for feature selection in the context of mixture models, such as the asymmetric Gaussian distribution (AGD) [62] and the asymmetric generalized Gaussian distribution (AGGD) [56]. However, these distributions are unbounded with a support region that extends across the set of real numbers. Real-life datasets are mostly digitized and have bounded support [18]. Therefore, we propose the bounded asymmetric generalized Gaussian distribution (BAGGD) to model the relevant features of each component in the mixture. The BAGGD distribution generalizes several different distribution classes, such as the impulsive, the Laplacian, the Gaussian, and the uniform distributions, to fit different shapes of observed bounded support, asymmetric, and non-Gaussian data. In order to define the bounded distribution proposed in this paper, the bounded support region $\tau_{kd}$ in R for each component is first defined for the following indicator function:

(7) $$H\left(X_{id}|k\right)=\left\{\begin{array}{cc} 1 & X_{id}\in \tau_{kd}\\ 0 & Otherwise \end{array}\right.$$

The bounded asymmetric generalized Gaussian probability density function for each D-dimensional data point is defined as follows:

(8) $$p\left(\vec{X_{i}}|\theta_{k}\right)=\prod_{d=1}^{D}\frac{\Psi \left(X_{id}|\theta_{kd}\right)H\left(X_{id}|k\right)}{\int_{\partial_{k}}\Psi \left(X_{id}|\theta_{kd}\right)dX}$$

The unbounded distribution $p(X_{id}|\theta_{kd})$ is the asymmetric generalized Gaussian distribution (AGGD). The symmetric and asymmetric generalized Gaussian distributions are defined in Equations (9) and (10), respectively.

(9) $$g\left(X_{id}|\mu_{kd},\sigma_{kd},\lambda_{kd}\right)=\frac{\lambda_{kd}{\left[\frac{\Gamma (3/\lambda_{kd})}{\Gamma (1/\lambda_{kd})}\right]}^{1/2}}{2\sigma_{kd}\Gamma \left(1/\lambda_{kd}\right)}exp\left[-A\left(\lambda_{kd}\right)|\frac{X_{id}-\mu_{kd}}{\sigma_{kd}}{|}^{\lambda_{kd}}\right]$$

(10) $$\begin{array}{cc} \; & \Psi \left(X_{id}|\theta_{kd}\right)=\left\{\begin{array}{cc} g_{1}\left(X_{id}|\theta_{kd}\right) & x<\mu_{kd}\\ g_{2}\left(X_{id}|\theta_{kd}\right) & x\geq \mu_{kd} \end{array}\right.=\frac{\lambda_{kd}{\left[\frac{\Gamma (3/\lambda_{kd})}{\Gamma (1/\lambda_{kd})}\right]}^{1/2}}{\left(\sigma_{l_{kd}}+\sigma_{r_{kd}}\right)\Gamma \left(1/\lambda_{kd}\right)}\\ & \times \left\{\begin{array}{cc} exp\left[-A\left(\lambda_{kd}\right){\left(\frac{\mu_{kd}-X_{id}}{\sigma_{r_{kd}}}\right)}^{\lambda_{kd}}\right] & X_{id}<\mu_{kd}\\ exp\left[-A\left(\lambda_{kd}\right){\left(\frac{X_{id}-\mu_{kd}}{\sigma_{l_{kd}}}\right)}^{\lambda_{kd}}\right] & X_{id}\geq \mu_{kd} \end{array}\right. \end{array}$$

where $A\left(\lambda_{kd}\right)={\left[\frac{\Gamma (3/\lambda_{kd})}{\Gamma (1/\lambda_{kd})}\right]}^{\lambda_{kd}/2}$; $\theta_{kd}=\left[\mu_{kd},\sigma_{l_{kd}},\sigma_{r_{kd}},\lambda_{kd}\right]$ represents the set of parameters that defines the AGGD for each mixture component. $\mu_{kd}$, $\sigma_{l_{kd}}$, $\sigma_{r_{kd}}$, and $\lambda_{kd}$ denote the mean, the left standard deviation, the right standard deviation, and the shape parameter of the AGGD, respectively. The shape parameter controls the distribution’s tails. The larger its value, the flatter the distribution at the mean; the smaller it is, the more peaked the distribution at the mean. The right and left variance combination allows the probability density function to be asymmetric or non-asymmetric. Thus, the proposed mixture model would consider the different shapes, asymmetry, and bounded support region of the smart meter data. Bounded distribution generalizes to all its special cases, including the bounded variants [18]. Thus, our proposed FSBAGGMM generalizes to a wide range of mixture models, including the bounded variants, as shown in Table 1. Additionally, we will demonstrate in Section 5 how the proposed FSBAGGMM can generalize feature selection models based on the asymmetric generalized Gaussian mixture, in addition to several specific mixture models in terms of modelling smart meter data.

## 4. Model Parameter Estimation and Selection

In this section, we will explain how the feature weights and the mixture model parameters are estimated for the modelling of the training data, in addition to the model selection criterion. We propose an approach to reveal the valid number of intrinsic clusters within a dataset using MML and estimate the proposed model’s parameters using EM.

### 4.1. Parameter Estimation Using the EM Algorithm

The mixture model’s parameters are optimized in parallel with the features’ weights in each iteration using the EM algorithm. The iterations of the EM algorithm produce a sequence of models with a non-decreasing log-likelihood. The parameters are optimized to achieve the maximum log-likelihood, and the log-likelihood function is expressed as follows:

(11) $$\begin{array}{cc} L\left(X,\Theta_{M},Z,\varphi \right)= & \sum_{i,k}p\left(Z_{i}=k|\vec{X_{i}}\right)\log p_{k}+\sum_{i,k}\sum_{d}\sum_{\varphi_{d}=0}^{1}p\left(Z_{i}=k,\varphi |\vec{X_{i}}\right)\\ & \times (\varphi_{d}(\log(p\left(X_{id}|\theta_{kd}\right)+\log w_{d})\\ \; & +\left(1-\varphi_{d}\right)\left(\log p\left(X_{id}|\beta_{d}\right)+\log\left(1-\omega_{d}\right)\right)) \end{array}$$

The EM algorithm has made the optimization process for mixture models feasible through an iterative process using Equation (11) instead of Equation (2). The conditional expected values $\gamma (Z_{jh})$ and $\hat{\omega_{d}}$ are given by Equations (12) and (13).

(12) $$p\left(Z_{i}=k|\vec{X_{i}},\Theta_{M}\right)=\gamma \left(Z_{ik}\right)=\frac{p_{k}\prod_{d=1}^{D}\zeta_{i,k,d}}{\sum_{j=1}^{K}p_{j}\prod_{d=1}^{D}\zeta_{i,j,d}}$$

(13) $$\begin{array}{c} {\hat{\omega }}_{d}=\frac{\sum_{i=1}^{N}\sum_{j=1}^{M}\frac{\omega_{d}p\left(X_{id}|\theta_{jd}\right)}{\zeta_{i,j,d}}\gamma \left(Z_{ij}\right)}{N} \end{array}$$

where $\zeta_{i,k,d}=\left[\omega_{d}p\left(X_{id}|\theta_{kd}\right)+\left(1-\omega_{d}\right)p\left(X_{id}|\beta_{d}\right)\right]$. The EM algorithm consists of a loop over two steps: the E-step and the M-step. They are performed repetitively until convergence. In the E-step, Equation (12) is evaluated using either the initial parameters or the parameters estimated in the M-step. In the M-step, the parameters of the next model in the sequence are estimated. Each estimated model in the sequence represents a better approximation of the distribution of the smart meter data. Due to the complicated nature of the BAGGD function, the gradient of the log-likelihood function (Equation (11)) with respect to each one of the parameters was non-linear, and a closed-form solution was not obtained; therefore, for these parameters, we used the Newton–Raphson method to approximate the update values, as demonstrated in the equations below. The partial derivatives obtained with respect to each of the parameters can be found in Appendix A. Thus, the M-step is implemented using the following equations:

(14) $$p_{k}=p\left(Z_{k}=1\right)=\frac{\sum_{i=1}^{N}p\left(k|\vec{X_{i}},\Theta_{M}\right)}{N}$$

(15) $$\begin{array}{c} \mu_{\hat{k}d}=\mu_{kd}-\left[{\left(\frac{\partial^{2}L\left(X,\Theta_{M},Z,\varphi \right)}{\partial \mu_{kd}^{2}}\right)}^{-1}\left(\frac{\partial L(X,\Theta_{M},Z,\varphi )}{\partial \mu_{kd}}\right)\right] \end{array}$$

(16) $$\begin{array}{c} \sigma_{{\hat{l}}_{kd}}=\sigma_{l_{kd}}-\left[{\left(\frac{\partial^{2}L\left(X,\Theta_{M},Z,\varphi \right)}{\partial \sigma_{l_{kd}}^{2}}\right)}^{-1}\left(\frac{\partial L(X,\Theta_{M},Z,\varphi )}{\partial \sigma_{l_{kd}}}\right)\right] \end{array}$$

(17) $$\begin{array}{c} \sigma_{{\hat{r}}_{kd}}=\sigma_{r_{kd}}-\left[{\left(\frac{\partial^{2}L\left(X,\Theta_{M},Z,\varphi \right)}{\partial \sigma_{r_{kd}}^{2}}\right)}^{-1}\left(\frac{\partial L(X,\Theta_{M},Z,\varphi )}{\partial \sigma_{r_{kd}}}\right)\right] \end{array}$$

(18) $$\begin{array}{c} \lambda_{\hat{k}d}=\lambda_{kd}-\left[{\left(\frac{\partial^{2}L\left(X,\Theta_{M},Z,\varphi \right)}{\partial \lambda_{kd}^{2}}\right)}^{-1}\left(\frac{\partial L(X,\Theta_{M},Z,\varphi )}{\partial \lambda_{kd}}\right)\right] \end{array}$$

(19) $$\begin{array}{c} {\hat{\eta }}_{d}=\frac{\sum_{i=1}^{N}\left[\frac{\left(1-\omega_{d}\right)p\left(X_{id}|\beta_{d}\right)}{\zeta_{i,k,d}}\gamma \left(Z_{ik}\right)\right]x_{id}}{\sum_{i=1}^{N}\sum_{j=1}^{M}\frac{\left(1-\omega_{d}\right)p\left(X_{id}|\beta_{d}\right)}{\zeta_{i,j,d}}\gamma \left(Z_{ij}\right)} \end{array}$$

(20) $$\begin{array}{c} {{\hat{\delta }}_{d}}^{2}=\frac{\sum_{i=1}^{N}\left[\frac{\left(1-\omega_{d}\right)p\left(X_{id}|\beta_{d}\right)}{\zeta_{i,k,d}}\gamma \left(Z_{ik}\right)\right]{\left(x_{id}-\eta_{d}\right)}^{2}}{\sum_{i=1}^{N}\sum_{j=1}^{M}\frac{\left(1-\omega_{d}\right)p\left(X_{id}|\beta_{d}\right)}{\zeta_{i,j,d}}\gamma \left(Z_{ij}\right)} \end{array}$$

### 4.2. Model Selection

Model selection involves selecting the best set of parameters that model the smart meter data. Among several candidate models, the model with the maximum log-likelihood may achieve the best fit to the data; however, it is not guaranteed to perform well on unseen data. In other words, model evaluation based on the log-likelihood exclusively could be misleading. In this section, we develop a model selection criterion to infer the true number of consumption profiles within a dataset in an unsupervised manner. The Minimum Message Length criterion [72,73] is an information-theory-based model selection method; it selects the best model among a list of candidate statistical models based on its capability of compressing a message containing the data. According to the MML criterion, the best model minimizes a message that consists of two parts: the first part encodes the model using prior knowledge about the model exclusively, and the second part encodes the data using the model. Given a list of candidate models, the following function is minimized to obtain the true number of intrinsic clusters within the data:

(21) $$\begin{array}{c} \mathrm{MessLens}\approx -\log p\left(\Theta_{M}\right)+\frac{c}{2}\left(1+\log\rho_{c}\right)+\frac{1}{2}\log\left|I\left(\Theta_{M}\right)\right|-\log p\left(X|\Theta_{M}\right) \end{array}$$

In Equation (21), the prior distribution is represented by $p(\Theta_{M})$, the determinant of the Fisher information matrix is represented by $\left|I(\Theta_{M})\right|$, and the model’s likelihood is represented by $p(X|\Theta_{M})$. The constant *c* is the total number of parameters; in this case, it is calculated as c=M+D+4DM+2D,c≥1. The term $\rho_{c}\in R^{c}$ represents the optimal quantization lattice constant [74]; the value of the constant is approximated with $\rho_{c}=\frac{1}{12}$ as the value of *c* changes across the list of candidate models [75]. The independence of the different clusters of parameters has been considered in this paper, which allows the factorization of the prior distribution and Fisher information matrix in Equation (21). Additionally, we approximate the determinant of the Fisher information matrix using the complete likelihood, and we consider the uninformative Jeffrey’s prior for the distribution of each group of parameters. Hence, in our case, the MML optimization objective function is calculated as follows:

(22) $$\begin{array}{cc} \mathrm{MessLens}\approx & \frac{c}{2}\left(1+\log\rho_{c}\right)+\frac{c}{2}\left(\log N\right)+2M\sum_{d=1}^{D}\log\omega_{d}+2d\sum_{k=1}^{M}\log p_{k}+\sum_{d=1}^{D}\log\left(1-\omega_{d}\right)\\ & -\log p(X|\Theta_{M}) \end{array}$$

Equation (22) is minimized with respect to several constraints [23], which are listed as follows: $0<p_{k}\leq 1$, $0\leq \omega_{d}\leq 1$, and $\sum_{j=1}^{M}p_{j}=1$. In the context of this model selection criterion, since we are estimating feature weights using the EM algorithm, Equations (23) and (24) are utilized alternatively to approximate the parameters ${\hat{p}}_{k}$ and ${\hat{\omega }}_{d}$, respectively, as follows:

(23) $$\begin{array}{c} {\hat{p}}_{k}=\frac{\max\left(\sum_{i=1}^{N}\sum_{j=1}^{M}\gamma \left(Z_{ij}\right)-2D,0\right)}{\sum_{j=1}^{M}\max\left(\sum_{i=1}^{N}\gamma \left(Z_{ij}\right)-2D,0\right)} \end{array}$$

(24) $$\begin{array}{c} {\hat{\omega }}_{d}=\frac{\max\left(\sum_{i=1}^{N}\sum_{j=1}^{M}\frac{\omega_{d}p\left(X_{id}|\theta_{jd}\right)}{\zeta_{i,j,d}}\gamma \left(Z_{ij}\right)-2M,0\right)}{T} \end{array}$$

(25) $$\begin{array}{cc} T & =\max\left(\sum_{i=1}^{N}\sum_{j=1}^{M}\frac{\omega_{d}p\left(X_{id}|\theta_{jd}\right)}{\zeta_{i,j,d}}\gamma \left(Z_{ij}\right)-2M,0\right)+\max\left(\sum_{i=1}^{N}\sum_{j=1}^{M}\frac{\left(1-\omega_{d}\right)p\left(X_{id}|\beta_{d}\right)}{\zeta_{i,j,d}}\gamma \left(Z_{ij}\right)-1,0\right) \end{array}$$

The Algorithm of Model Selection and Model Parameter Estimation

Algorithm 1 describes how to perform model selection and feature selection using the MML criterion and model parameter estimation using the EM algorithm.
**Algorithm 1:** Unsupervised FSBAGGMM1:**While**$M<M_{max}$**do**2:     Initialize $\Theta_{M}$K-means clustering results are used to initialize the parameters $\left(\pi_{1},\ldots ,\pi_{M},\vec{\mu_{1}},\ldots ,\vec{\mu_{M}},\vec{\sigma_{l_{1}}},\ldots ,\vec{\sigma_{l_{M}}},\vec{\sigma_{r_{1}}},\ldots ,\vec{\sigma_{r_{M}},\lambda_{1},\ldots ,\lambda_{M}}\right)$.For each cluster *k*, each element of the parameter vector $\vec{\lambda_{k}}$ is set to the value 2.Initialize the background Gaussian distribution parameter set $\vec{\beta }$ using the following equations for all the dimensions, where d∈{1,…,D}:
(26)$$\eta_{d}=\frac{1}{N}\sum_{i=1}^{N}X_{id}$$
(27)$$\delta_{d}^{2}=\frac{1}{N}\sum_{i=1}^{N}{\left(X_{id}-\eta_{d}\right)}^{2}$$3:     Implement the E-step.For each cluster *k*, compute the bounded support region $\vec{\tau_{k}}=\left(\tau_{1},\ldots ,\tau_{D}\right)$.Evaluate Equation (12).**if** $\omega_{d}=0$ **Then** $p(X_{id}|\theta_{kd})=0$**if** $\omega_{d}=1$ **Then** $p(X_{id}|\beta_{d})=0$4:     Implement the M-step using Equations (15) through (20), (23), and (24).5:     **if** $p{\left(X|\Theta \right)}^{\iota +1}-p{\left(X|\Theta \right)}^{\iota }<\epsilon$ **then**Calculate the message length using Equation (22).

### 4.3. Implementation with HPC

The advancements in computational methodologies have played a pivotal role in addressing the challenges of data processing, especially in the realm of smart meters. Given the magnitude and intricacy of the data generated by these meters, traditional computing methods often fall short. This necessitated the exploration and implementation of our algorithm via HPC.

Our choice of HPC was rooted in its inherent capability to expediently process large volumes of data. For the clustering task at hand, HPC provided the computational agility required to analyze vast datasets from smart meters swiftly. By leveraging the parallel processing capabilities of HPC, we could achieve a significant reduction in computation time, while ensuring the consistency and accuracy of our clustering results.

Edge cloud computing stands at the forefront of modern computational paradigms, emphasizing on-the-spot processing to facilitate real-time decision-making. With the integration of HPC in edge settings, we foresee several advantages.

- Enhanced Speed and Efficiency: By employing HPC at the edge, data from smart meters can be processed locally, resulting in quicker analytics and response times. This is especially crucial for utility programs that require timely information, such as demand response and energy efficiency initiatives.
- Scalability: As the deployment of smart meters expands, the amount of data to be processed will proportionally increase. HPC can readily handle this surge, ensuring that the system can scale without compromising on performance.
- Real-Time Analytics for Utility Programs: HPC, coupled with edge cloud computing, can power real-time analytics. For instance, utility providers can swiftly analyze consumption patterns and roll out demand response strategies almost instantaneously. This not only enhances grid reliability but also aids in optimizing energy consumption and costs for consumers.

## 5. Experimental Results

In this section, we will validate the performance of the MML model selection criterion and the proposed FSBAGGMM using two synthetic and real-life smart meter datasets within the application of household energy consumption segmentation. The first real-life dataset was recorded by the Commission for Energy Regulation (CER) and made accessible for researchers by the Irish Social Science Data Archive (ISSDA) [4]. The dataset consists of smart meter data gathered from more than 6000 Irish energy consumers from 14 July 2009 to 31 December 2010. The energy consumption is recorded in kWh with an interval of half an hour. This dataset has two types of energy consumers: residential and small to medium enterprises. As stated earlier, we are interested in analyzing the energy consumption of residential energy consumers only. Therefore, 3639 Irish residential energy consumers remain for analysis after data cleaning. Each residential consumer is assigned six different tariffs (E, A, D, C, B, and W). The second real-life smart meter dataset consists of smart meter data collected from 5567 residential homes in London. The data were collected by the UK Power Networks led by the Low Carbon London Project between November 2011 and February 2014 [6]. The energy consumption is recorded in kWh with an interval of half an hour. After data cleaning, observations of 3891 household energy consumers within the year 2013 are used to analyze this experiment. The residential energy consumers in this dataset are subjected to two types of tariffs. The first type is the dynamic time of use (ToU), where the energy consumption prices vary as follows: high (67.20 pence/kWh), low (3.99 pence/kWh), or normal (11.76 pence/kWh). The second type is the standard (std), where the consumers pay a flat rate of 14.228 pence/kWh. Additionally, the energy consumers in this dataset belong to five different geo-demographic groups.

The application considered in this paper aims to segment energy consumers given their load curve. We use characteristic load profiles to find the optimal number of energy consumption clusters with similar consumption patterns and determine the cluster membership of every load curve given in the training dataset. Utility companies can use accurate energy-consumer-type identification to make correct decisions regarding the investments in load-shifting campaigns to prevent over- or under-dimensioning linked to the peak energy demand. Several performance evaluation metrics [64] are used in this paper. They are defined as follows.

DI [76]: Dunn’s index is a model performance evaluation metric that is calculated using the minimum ratio between the closest distance of two observations of different clusters and the largest distance between two observations in the same cluster. This index is maximized for the best clustering and it is defined as follows:

(28) $$\mathrm{DI}=\frac{\min_{A\in M}\left\{\min_{B\in M,B\neq A}\left\{\phi \left(A,B\right)\right\}\right\}}{\max_{A\in M}\left\{\Pi \left(A\right)\right\}}$$

(29) $$\phi \left(A,B\right)=\min_{{\vec{X}}_{i}\in A,{\vec{Y}}_{j}\in B}\left\{d({\vec{X}}_{i},{\vec{Y}}_{j})\right\}$$

(30) $$\Pi \left(A\right)=\max_{{\vec{X}}_{i},{\vec{X}}_{j}\in A}\left\{d({\vec{X}}_{i},{\vec{X}}_{j})\right\}$$

where *d* denotes the distance or the similarity function, ϕ(A,B) denotes the minimum distance between two observations that each belong to either cluster A or B, and M denotes the set of clusters.

EoE [31]: The *entropy of eigenvalues* is an entropy-based clustering performance measure; it is obtained from the eigenvalue analysis of the correlation matrix calculated using raw smart meter data. The index is calculated using the correlation between representative time series of different clusters and the correlation between different time series within each cluster. The EoE index is calculated using the following equation:

(31) $$\mathrm{EoE}=\frac{SM_{B}}{\sum_{k}^{K}\frac{N_{k}}{N}SM_{wk}}$$

The SM similarity is a normalized average information measure; the larger it is, the greater the similarity. The term $SM_{b}$ represents the normalized entropy of eigenvalues obtained from the correlation matrix between different clusters, and $SM_{wk}$ represents the normalized entropy of eigenvalues obtained from the correlation matrix between time series in each cluster *k*. In an ideal clustering, EoE is a small value consisting of high similarity between time series within each cluster and low similarity between representative time series of different clusters.

S [77]: The silhouette score is a model evaluation measure that is concerned with calculating a score for each observation in the training dataset. The measure calculates the overall evaluation by computing the average score for all the dataset observations. The metric is maximized for better clustering and is defined in the following equation:

(32) $$s\left(x_{i}\right)=\frac{b\left(x_{i}\right)-a\left(x_{i}\right)}{max\{a\left(x_{i}\right),b\left(x_{i}\right)\}}$$

where $a(x_{i})$ represents the average dissimilarity of the data point $x_{i}$ to all the other data points within the same cluster. $b(x_{i})$ represents the minimum average dissimilarity of data point $x_{i}$ to data points existing in a cluster different from the data point’s cluster.

CH [78]: The Calinski–Harabasz index is a model performance evaluation index; the measure calculates the ratio between the inter-cluster variance and the intra-cluster variance. This measure is maximized for better clustering and is defined as follows:

(33) $$\mathrm{CH}=\frac{N-K}{K-1}\frac{\sum_{k=1}^{K}\left(N_{k}d\left(c_{k},\bar{c}\right)\right)}{\sum_{k=1}^{K}\sum_{i=1}^{N_{k}}d\left({\vec{X}}_{i},c_{k}\right)}$$

where $N_{k}$ is the number of observations predicted to belong to cluster *k*, $c_{k}$ denotes the centroid of class *k*, $\bar{c}$ denotes the global centroid of all the clusters, and *d* denotes the distance or the similarity function.

DB [79]: The Davies–Bouldin index is a model performance evaluation measure; it calculates the ratio of intra-cluster distances to inter-cluster distances for each possible pair of clusters. The maximum ratio calculated for each pair of clusters is considered in a summation. The summation result is divided by the total number of clusters to obtain the metric’s value. This measure is minimized for better clustering, and it is defined as follows:

(34) $$\mathrm{DB}=\frac{1}{k}\sum_{A\in M}\max_{B\in M,B\neq A}\left\{\frac{O(A)+O(B)}{d(c_{A},c_{B})}\right\}$$

(35) $$O\left(A\right)=\frac{1}{\varrho (A)}\sum_{{\vec{X}}_{i}\in A}d\left({\vec{X}}_{i},c_{A}\right)$$

where ϱ(A) denotes the cardinality of cluster A, k denotes the number of components enforced by the mixture model, M denotes the set of clusters, $c_{A}$ denotes the centroid of class *A*, and *d* denotes the distance or the similarity function. M has k elements.

GOF [80]: The goodness of fit statistic value measures the model’s fitting accuracy and it is calculated as follows:

(36) $$\mathrm{GOF}=\sum_{i=1}^{N}\frac{{\left(\Upsilon \left({\vec{X}}_{i}\right)-\Omega \left({\vec{X}}_{i}\right)\right)}^{2}}{\Omega ({\vec{X}}_{i})}$$

where $\Upsilon ({\vec{X}}_{i})$ and $\Omega ({\vec{X}}_{i})$ represent the empirical and the expected frequencies of the observation ${\vec{X}}_{i}$, respectively. The indices ACC, TPR, PPV, TNR, NPV, FPR, FNR, and FDR represent the average accuracy, average true positive rate, positive predictive value, true negative rate, negative predictive value, false positive rate, false negative rate, and false discovery rate, respectively. They are defined as follows:

(37) $$\mathrm{TPR}=\frac{1}{M}\sum_{k=1}^{M}\frac{{\mathrm{TP}}_{k}}{{\mathrm{TP}}_{k}+{\mathrm{FN}}_{k}}$$

(38) $$\mathrm{TNR}=\frac{1}{M}\sum_{k=1}^{M}\frac{{\mathrm{TN}}_{k}}{{\mathrm{TN}}_{k}+{\mathrm{FP}}_{k}}$$

(39) $$\mathrm{PPV}=\frac{1}{M}\sum_{k=1}^{M}\frac{{\mathrm{TP}}_{k}}{{\mathrm{TP}}_{k}+{\mathrm{FP}}_{k}}$$

(40) $$\mathrm{NPV}=\frac{1}{M}\sum_{k=1}^{M}\frac{{\mathrm{TN}}_{k}}{{\mathrm{TN}}_{k}+{\mathrm{FN}}_{k}}$$

(41) $$\mathrm{FPR}=\frac{1}{M}\sum_{k=1}^{M}\frac{{\mathrm{FP}}_{k}}{{\mathrm{FP}}_{k}+{\mathrm{TN}}_{k}}$$

(42) $$\mathrm{FNR}=\frac{1}{M}\sum_{k=1}^{M}\frac{{\mathrm{FN}}_{k}}{{\mathrm{TP}}_{k}+{\mathrm{FN}}_{k}}$$

(43) $$\mathrm{FDR}=\frac{1}{M}\sum_{k=1}^{M}\frac{{\mathrm{FP}}_{k}}{{\mathrm{TP}}_{k}+{\mathrm{FP}}_{k}}$$

(44) $$\mathrm{ACC}=\frac{1}{M}\sum_{k=1}^{M}\frac{{\mathrm{TP}}_{k}+{\mathrm{TN}}_{k}}{{\mathrm{TP}}_{k}+{\mathrm{FP}}_{k}+{\mathrm{FN}}_{k}+{\mathrm{TN}}_{k}}$$

where ${\mathrm{TP}}_{k}$, ${\mathrm{FP}}_{k}$, ${\mathrm{TN}}_{k}$, and ${\mathrm{FN}}_{k}$ denote the number of true positives, false positives, true negatives, and false negatives, respectively, for the cluster *k*. In order to compute the metrics explained in Equations (37)–(44), cluster *k* labels are considered a positive class and all the remaining cluster labels are considered a negative class. MCC represents the Matthews correlation coefficient evaluation metric [81].

The AIC and BIC are probabilistic model selection methods [82] that attempt to select the model with the best performance while taking into consideration its complexity (by adding a complexity-related penalty). Unlike probabilistic model selection criteria, performance metrics select models with no regard to their complexity. The distinct probabilistic model selection criteria used in this paper originate from different fields of study. The AIC is derived from the frequentist framework, while the BIC is derived from Bayesian probability and inference. Compared to the BIC, the AIC emphasizes the model performance and penalizes complex models less, making it prone to selecting overfitted models. In comparison to the AIC, the BIC attempts to penalize candidate models more for their complexity. The AIC and BIC model selection criteria statistics for each candidate model are computed as follows:

(45) BIC=2log(L(Θ))+κlog(N)

(46) $$AIC=\frac{-2}{N}\log\left(L\left(\Theta \right)\right)+2*\frac{\kappa }{N}$$

where L(Θ) is the likelihood function estimate given a set of parameters Θ, κ represents the number of free parameters, and *N* represents the number of observations. As *N* approaches infinity, the BIC criterion is more likely to select the candidate model with the true number of intrinsic clusters. The candidate model with the lowest AIC and BIC is selected for both model selection criteria.

In the upcoming sections, the performance of the proposed model is compared to specific mixture models such as the BAGGMM, the AGGMM, and the FSAGGMM. Model selection using the proposed model is performed using the MML model selection criterion and compared against specific model selection methods such as the BIC and AIC, and model selection methods using performance measures, such as Dunn’s index (DI) and the entropy of eigenvalues (EoE).

### 5.1. Synthetic Data

As a first stage, synthetic datasets are used to validate the proposed mixture model and its model selection method. We propose using a 49-dimensional dataset, which imitates a real-life smart meter dataset by representing each energy consumer with a load curve. In order to generate the synthetic datasets used in this paper, the following steps were followed.

1. For each energy consumer in the real-life dataset, only the first 49 smart meter observations are considered.
2. The Gaussian mixture model is used to cluster the data into a specific number of clusters. The mean of each cluster is considered a consumption profile.
3. Each consumption profile inferred from the previous step is summed with instances generated by Gaussian white noise using five different sets of parameters to form the observations of the synthetic dataset.

In other words, the origin of each cluster of observations within the synthetic datasets used in this paper is an actual energy consumption profile concluded from a real dataset.

The data-generating process delineated above provides a systematic approach to crafting synthetic datasets with asymmetric class distributions and varied shapes. By grounding the data in real consumption profiles and subsequently introducing variations via Gaussian white noise, the process ensures a rich diversity of data shapes. This diversity serves as a rigorous testing ground to evaluate the flexibility and robustness of the proposed mixture model, effectively challenging its capability to adapt and accurately represent varied data structures.

**Figure 2:**
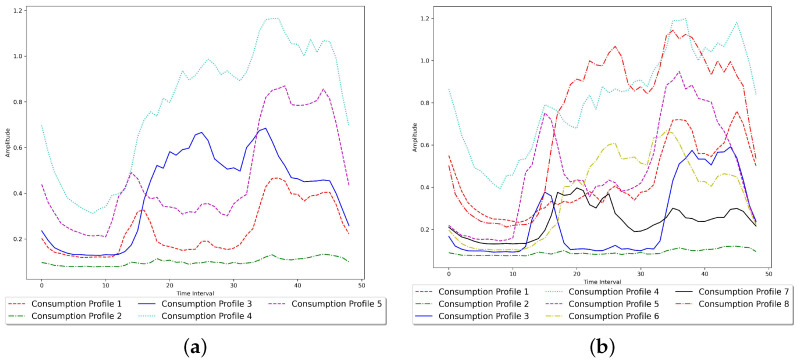
Consumption profiles used to generate the synthetic datasets. (**a**) First synthetic dataset. (**b**) Second synthetic dataset.

The first dataset consists of five clusters. The five real-life consumption profiles used to generate the first dataset are demonstrated in Figure 2a. The count of the observations generated for each energy consumption profile using the distinct Gaussian white noise parameters is shown in Table 2. The clustering results of our proposed model are evaluated using several performance measures and compared against the clustering performance of specific mixture models, as shown in Table 3 and Table 4. As an illustrative example of the data generation process, 378 observations of the first dataset are generated by summing the white noise vector generated using the parameter set (μ=0.001;σ=0.2) of the multivariate Gaussian white noise with the vector of “Consumption Profile 1”.

Our model selection approach successfully infers the correct number of components within this dataset, as demonstrated in Table 5. MML outperforms specific model selection methods using the clustering results obtained from each instance of our proposed model.

**Figure 3:**
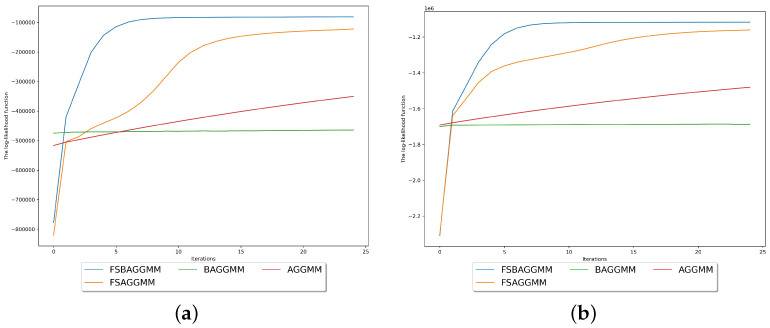
Mixture model’s log-likelihood function demonstration during the clustering of the synthetic datasets. (**a**) First synthetic dataset. (**b**) Second synthetic dataset.

Figure 3a demonstrates the maximum log-likelihood achieved by clustering the data using the proposed model in comparison with specific mixture models. The proposed model achieves the best fit of the training data by achieving the best performance according to all the performance metrics used in this experiment and by reaching the highest log-likelihood. 

The second dataset consists of eight clusters. The eight real-life consumption profiles used to generate this dataset are demonstrated in Figure 2b. Our model selection approach successfully infers the correct number of components within this dataset, as demonstrated in Table 6. The count of the observations generated for each energy consumption profile using the distinct Gaussian white noise parameters is shown in Table 7. MML chooses the proposed model’s instance with a component count equal to the ground truth, outperforming specific model selection methods used in this comparison. The proposed model fits the data better than all the mixture models used in the comparison by achieving the highest maximum log-likelihood, as demonstrated in Figure 3b. According to all the performance metrics used in this experiment, the proposed model also outperforms the mixture models selected for the comparison, as shown in Table 8 and Table 9.

### 5.2. Real-Life Smart Meter Data

#### 5.2.1. The Commission for Energy Regulation Smart Meter Data

In this section, we investigate the performance of our proposed model using the first real-life smart meter dataset. As mentioned earlier, the dataset that we consider has smart meter observations from 3639 Irish energy consumers. Each consumer has 25,728 electricity usage readings that are recorded in kilowatt-hours. In order to summarize and preserve the information within the numerous features representing each energy consumer, PCA is used for feature extraction in this experiment. Several datasets with a different number of features are considered within the range between 50 and 250. Due to the low reconstruction error, the dataset with 250 features is favoured for this experiment.

**Figure 4:**
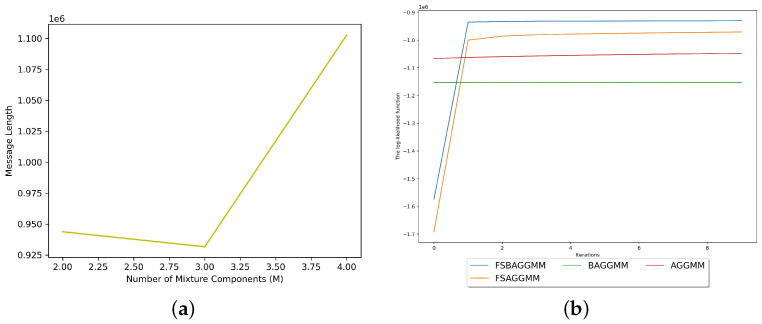
The mixture models’ performance information during the clustering of the first real-life smart meter data. (**a**) Selection of the optimal number of mixture components using MML and the proposed model. (**b**) The log-likelihood functions of the mixture models used in the comparison.

We used the dataset as an input to three different instances of our proposed model. Each instance had a different number of mixture components within the range M=[2,4]. The model selection algorithm concluded that the minimum value calculated using its objective function was obtained while using the model instance with three components, as shown in Figure 4a. Table 10 demonstrates the optimal number of clusters concluded by each model selection criterion used in comparison with MML. In addition to the fact that our derived model selection criterion infers the correct number of clusters in solid experiments using synthetic data, the AIC and BIC also agree that the true number of clusters is three in this experiment.

Figure 4b demonstrates the log-likelihood trail for each mixture model used in the comparison within this experiment. As observed, the proposed model converged to the highest log-likelihood, indicating a better fit to the training dataset. The clustering evaluation of the proposed model for the concluded optimal number of clusters is demonstrated in Table 11 in comparison with specific mixture models. As demonstrated, our proposed model achieves the best clustering performance according to all the evaluation measures used in the comparison.

**Figure 5:**
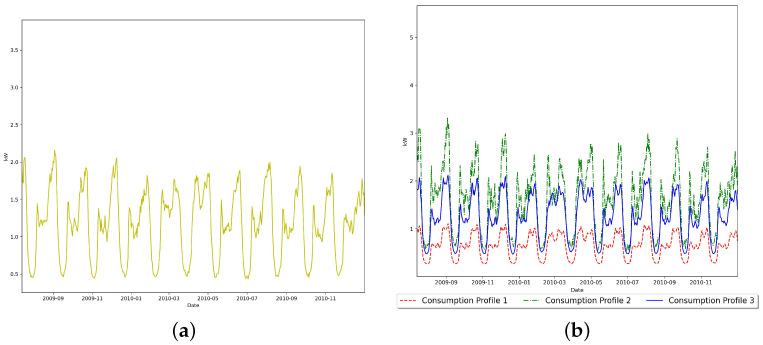
Household energy consumption segmentation demonstration of the first real-life smart meter dataset. (**a**) The average demand of all the energy consumers starting from 14 July 2009 to 31 December 2010. (**b**) The average demand of the optimal energy consumption clusters from 14 July 2009 to 31 December 2010.

**Figure 6:**
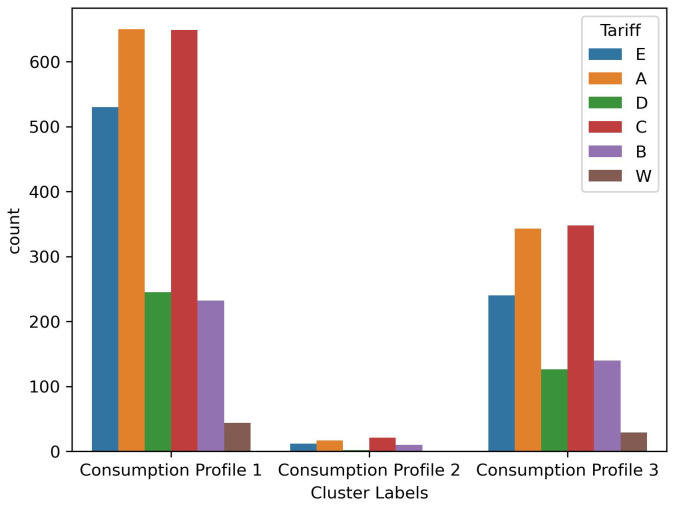
Number of energy consumers in each cluster.

As mentioned earlier, we determined the true number of clusters using MML and achieved the best clustering result using our proposed mixture model. Since this is an implementation of a real-life application, it is necessary to analyze the resulting clusters to understand further the energy consumption patterns of each consumption trend discovered. Figure 5a demonstrates the average power demand of all the energy consumers without clustering. Comparatively, we demonstrate the average power demand of each energy consumer cluster in Figure 5b. For all the time intervals available in the dataset, as observed, the responsibility of each energy consumption pattern to the overall average power demand can be determined. The proposed model can determine the consumer’s contribution to each consumption profile and which the consumer is mostly following. Table 12 demonstrates the ratio of the count of energy consumers in each cluster to the total count of energy consumers in the dataset; the table also demonstrates the consumption responsibility of each consumer cluster to the total average energy consumption in the year 2010. Additionally, the real-life dataset that we use in this experiment provides the tariff assigned for each energy consumer. We have discovered that the tariff types are distributed almost identically across the resulting clusters, as shown in Figure 6, which indicates that the tariff type does not influence the consumer’s electrical usage pattern.

#### 5.2.2. The UK Power Networks Smart Meter Data

In this section, we validate the performance of our proposed model using the second real-life smart meter dataset. As mentioned earlier, the dataset that we consider in this experiment has smart meter observations from 3891 household energy consumers that are located in London. Each consumer has 17,520 electricity usage readings that are recorded in kilowatt-hours. In order to summarize the information included in the load curve of each energy consumer, we have extracted nine features. Following [32], seven features are extracted after the definition of four key time periods and they are denoted by t∈{1,2,3,4}. The overnight time period (t=1) is defined between 10:30 p.m. and 6:30 a.m., the breakfast time period (t=2) is defined between 6:30 a.m. and 9:00 a.m., the daytime period (t=3) is defined between 9:00 a.m. and 3:30 p.m., and the evening time period (t=4) is defined between 3:30 p.m. and 10:30 p.m. Based on the four previously explained prominent time periods, seven features are extracted from the smart meter data to summarize the representation of energy consumers, and they are calculated as follows.

- **${\mathrm{RAP}}_{t}$** denotes the relative average power for time period (t) over the entire year; it is defined as follows:
  (47) $${\mathrm{RAP}}_{t}=\frac{{\mathrm{AP}}_{t}}{\mathrm{DAP}},t=1,2,3,4$$
- the **meanSTD** denotes the mean relative standard deviation of the average power used over the entire year; it is defined as follows:
  (48) $$\mathrm{Mean}\mathrm{STD}=\frac{1}{4}\sum_{t=1}^{4}\frac{\sigma_{t}}{{\mathrm{AP}}_{t}}$$
- The **seasonal score** is defined as follows:
  (49) $$\mathrm{Seasonal}\mathrm{Score}=\sum_{t=1}^{4}\frac{|AP_{t}^{W}-AP_{t}^{S}|}{AP_{t}}$$
- The weekend vs. weekday difference score (**WD-WE diff. score**) is calculated as follows:

(50) $$\mathrm{WD}-\mathrm{WE}\mathrm{diff}.\mathrm{Score}=\sum_{i=1}^{4}\frac{|{\mathrm{AP}}_{t}^{\mathrm{WD}}-{\mathrm{AP}}_{t}^{\mathrm{WE}}|}{{\mathrm{AP}}_{t}}$$

where ${\mathrm{AP}}_{t}$, and $\sigma_{t}$ represent the average power used by the specific consumer and its corresponding standard deviation in the time period (*t*), respectively, over all the available smart meter data. DAP represents the average daily power used by the specific consumer throughout the available smart meter data. $AP_{t}^{W}$ and $AP_{t}^{S}$ represent the average power used by the specific consumer in the time period (*t*) throughout winter and summer, respectively. ${\mathrm{AP}}_{t}^{\mathrm{WD}}$, and ${\mathrm{AP}}_{t}^{\mathrm{WE}}$ represent the average power used by the specific consumer in the time period (*t*) throughout the weekdays and weekends, respectively, for the available data. Finally, the eighth and the ninth features represent the consumer’s tariff and geo-demographic group, respectively.

We have determined the optimal number of clusters for our proposed model using the MML model selection criterion, similarly to our previous experiments. Among five candidate FSBAGGMM models of mixture components within the range [2, 6], the model instance with four components achieved the minimum message length.

Most of the model selection methods used in the comparison demonstrated in Table 13 agree on the optimal number of mixture components. Therefore, the data were clustered into four clusters using our proposed model, and the clustering performance evaluation was compared against specific mixture models. Table 14 demonstrates how our proposed mixture model has been able to outperform the different mixture models used in the comparison using six different performance metrics.

**Figure 7:**
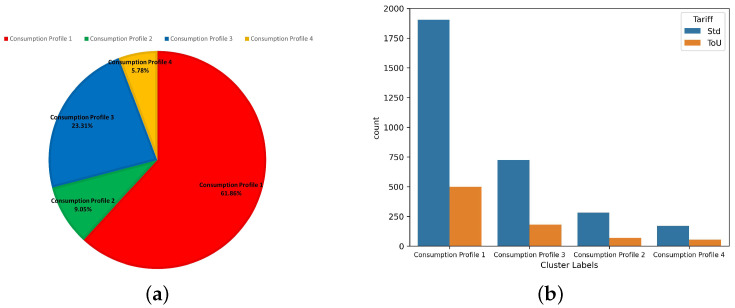
The UK Power Networks smart meter data clusters information. (**a**) Percentage of energy consumers in each cluster. (**b**) The distribution of tariffs across the resulting clusters.

As shown in Figure 7b, the categorical feature representing the tariff for each energy consumer has an almost identical distribution across the clusters obtained using our proposed mixture model, having little to no influence on the energy consumption behaviour. Nevertheless, as demonstrated by the CH score in Table 14, our proposed model has achieved clusters with relatively small intra-cluster (within clusters) variance and relatively large inter-cluster (between clusters) variance. Additionally, the minimum number of members within the clusters achieved using the FSBAGGMM is 225 energy consumers, as demonstrated in Figure 7a. Additionally, Table 15 demonstrates the average values of several features for the inferred household energy consumer clusters.

Since the smart meter data have been modelled successfully, the proposed model is capable of identifying energy consumer clusters that are suitable for demand reduction initiatives within several utility programs [2]. As an example, Table 15 demonstrates that the first cluster has a relatively high evening RAP with a relatively low mean STD, seasonal score, and WD-WE difference score. The power demand of energy consumers exhibiting energy consumption patterns similar to the first cluster could be lowered by implementing storage devices. The third and fourth clusters’ energy consumption patterns exhibit relatively low variability in demand, as represented by the mean STD and WD-WE difference score, while exhibiting a relatively high seasonal difference in power demand, as represented by the seasonal score. Such households could be offered non-electric or more efficient heating systems to reduce the winter demand.

## 6. Discussion

In this paper, we have presented an expectation-maximization algorithm within the MML criterion to optimize the parameters of the bounded asymmetric generalized Gaussian mixture model and to find the optimal number of consumption profiles and the optimal subset of features simultaneously. Our approach assumes that the data arise from a mixture of bounded asymmetric generalized Gaussian distributions. The final results demonstrate that the load curve of an individual energy consumer shows a probabilistic association with each class, indicating which pattern of electricity use is more or less likely to be used within a household. Therefore, it is possible to categorize households and how they consume energy using our proposed model.

Prior works in household energy consumption segmentation unrealistically approach model selection and feature selection as independent problems. Our approach successfully achieves the discovery of the true number of energy consumption profiles and the determination of the optimal set of data attributes to be used for modelling in our proposed mixture model in a single optimization process and avoids running the EM algorithm many times.

Clustering synthetically generated smart meter data with a ground truth cluster size, our proposed algorithm has outperformed most of the existing model selection approaches. In the same experiment, the proposed model correctly models the first and the second synthetic smart meter data with high accuracy of 95.569% and 91.856%, respectively. Similarly, our algorithm has also determined the optimal number of clusters in both datasets in experiments involving real-life data, and the proposed model outperforms all the mixture models used in the comparison, as demonstrated by all the utilized performance metrics. Thus, the superiority of the proposed algorithm in modelling smart meter data with different feature extraction methods over all the state-of-the-art clustering algorithms used in the comparison is proven.

Privacy and security concerns loom large in the realm of smart meter data analytics. Fortunately, the datasets employed in our research have been thoughtfully curated, with a paramount emphasis on safeguarding the privacy of individuals whose households are equipped with smart meters. These datasets meticulously exclude any information that might compromise the privacy of the participants while providing valuable insights for research. We have underscored in our research paper, particularly in the Results section, that the conventional categorization, carried out prior to any consumption data observation, is fundamentally ineffective. Respecting individuals’ privacy is not only an ethical imperative but also a fundamental human right. Remarkably, our proposed mixture model navigates this privacy-centric landscape adeptly. It uncovers the underlying data distribution and identifies energy consumption patterns without the need for additional, potentially intrusive information. This privacy-preserving approach aligns with the broader scientific quest for generalization and effectiveness in solutions that refrain from privacy invasion. Furthermore, our experiments with real-life datasets, which encompassed features such as tariff and geo-demographic groups, yielded intriguing results. These attributes, often considered vital, were deemed unimportant by our meticulous feature selection approach. This underscores our commitment to privacy and our ability to derive meaningful insights without resorting to invasive practices.

Finally, our implementation underscores a promising synergy between HPC and edge cloud computing, especially in the realm of smart meter data processing. As we progress towards a more interconnected and data-centric world, the amalgamation of these technologies will prove indispensable in sculpting the future of energy management and utility programs.

## 7. Conclusions

Our approach to analyzing real-life smart meter data is effective in determining households that are suitable for demand reduction initiatives such as DR and EE, thus providing the opportunity for utility companies to adopt environmentally friendly and cost-effective technologies.

The application addressed in this paper is well suited for an unsupervised approach, especially given the absence of ground truth labels. However, many applications would benefit from supervised or semi-supervised machine learning solutions. A limitation of the current learning framework presented in this paper is its inability to leverage ground truth labels. Recognizing this as a crucial area of improvement, future work could involve proposing a learning method for the mixture model that incorporates these labels to optimize the model parameters. 

## Figures and Tables

**Table 1:** FSBAGGMM special cases.

Special Case	Required Change in FSBAGGMM Parameters
Feature selection model based on	
the Asymmetric Generalized Gaussian Mixture (FSAGGMM) [56]	$H(X_{id}|k)=1$
Feature selection model based on	
the Bounded Asymmetric Gaussian Mixture (FSBAGMM)	$\lambda_{kd}=2$
Feature selection model based on	
the Asymmetric Gaussian Mixture (FSAGMM) [62]	$H\left(X_{id}|k\right)=1,\;\lambda_{kd}=2$
Feature selection model based on	
the Bounded Generalized Gaussian Mixture (FSBGGMM)	$\sigma_{r_{kd}}=\sigma_{l_{kd}}$
Feature selection model based on	
the Generalized Gaussian Mixture (FSGGMM)	$\sigma_{r_{kd}}=\sigma_{l_{kd},\;H\left(X_{id}|k\right)=1}$
Feature selection model based on	
the Bounded Gaussian Mixture (FSBGMM)	$\sigma_{r_{kd}}=\sigma_{l_{kd}},\;\lambda_{kd}=2$
Feature selection model based on	
the Gaussian Mixture (FSGMM)	$\sigma_{r_{kd}}=\sigma_{l_{kd}},\;\lambda_{kd}=2,\;H\left(X_{id}|k\right)=1$
Feature selection model based on	
the Bounded Laplace Mixture (FSBLMM)	$\sigma_{r_{kd}}=\sigma_{l_{kd}},\;\lambda_{kd}=1$
Feature selection model based on	
the Laplace Mixture (FSLMM)	$\sigma_{r_{kd}}=\sigma_{l_{kd}},\lambda_{kd}=1,\;H\left(X_{id}|k\right)=1$
Asymmetric Generalized Gaussian Mixture Model (AGGMM) [55]	$H\left(X_{id}|k\right)=1,\;\omega_{d}=1$
Bounded Asymmetric Gaussian Mixture Model (BAGMM)	$\lambda_{kd}=2,\;\omega_{d}=1$
Asymmetric Gaussian Mixture Model (AGMM) [69]	$H\left(X_{id}|k\right)=1,\;\lambda_{kd}=2,\;\omega_{d}=1$
Bounded Generalized Gaussian Mixture Model (BGGMM) [18]	$\sigma_{r_{kd}}=\sigma_{l_{kd}},\;\omega_{d}=1$
Generalized Gaussian Mixture Model (GGMM) [49]	$\sigma_{r_{kd}}=\sigma_{l_{kd},\;H\left(X_{id}|k\right)=1},\;\omega_{d}=1$
Bounded Gaussian Mixture Model (BGMM) [70]	$\sigma_{r_{kd}}=\sigma_{l_{kd}},\;\lambda_{kd}=2,\;\omega_{d}=1$
Gaussian Mixture Model (GMM)	$\sigma_{r_{kd}}=\sigma_{l_{kd}},\;\lambda_{kd}=2,\;H\left(X_{id}|k\right)=1,\;\omega_{d}=1$
Bounded Laplace Mixture Model (BLMM) [71]	$\sigma_{r_{kd}}=\sigma_{l_{kd}},\;\lambda_{kd}=1,\;\omega_{d}=1$
Laplace Mixture Model (LMM)	$\sigma_{r_{kd}}=\sigma_{l_{kd}},\;\lambda_{kd}=1,\;H\left(X_{id}|k\right)=1,\;\omega_{d}=1$

**Table 2:** Count of observations generated for the first synthetic dataset.

Gaussian White Noise Parameters	Profile 1	Profile 2	Profile 3	Profile 4	Profile 5
μ = 0.001; σ = 0.2	378	370	379	371	382
μ = 0.01; σ = 0.2	349	364	356	356	355
μ = 0.1; σ = 0.2	352	360	361	359	348
μ = 0.05; σ = 0.3	354	358	359	356	353
μ = 0.01; σ = 0.3	365	353	357	350	355

**Table 3:** Mixture models’ clustering performance evaluation using the first synthetic dataset.

Performance Index (%)	FSBAGGMM	FSAGGMM	BAGGMM	AGGMM
ACC	95.569	94.338	85.458	82.804
TPR/Recall	88.935	85.836	63.589	56.953
PPV/Precision	89.458	88.149	74.838	70.500
MCC	86.291	82.921	58.170	51.104
F1-Score	88.922	85.844	63.644	57.011
TNR	97.231	96.461	90.906	89.245
NPV	97.263	96.591	92.128	90.942
FPR	2.769	3.539	9.094	10.755
FNR	11.065	14.164	36.411	43.047
FDR	10.542	11.851	25.162	29.500

**Table 4:** Mixture models’ clustering performance evaluation using the first synthetic dataset.

Performance Index	Optimal Performance Indicator	FSBAGGMM	FSAGGMM	BAGGMM	AGGMM
GOF	Minimum	3870.683	7261.083	16,397.633	17,765.500
CH	Maximum	2081.868	2046.444	1594.215	1405.947
S	Maximum	0.107	0.100	0.023	−0.016
DB	Minimum	2.549	2.623	2.661	2.503
DI	Maximum	0.224	0.219	0.209	0.209
Xie and Benie Index	Minimum	1.871	1.881	2.446	2.698
Fowlkes Mallows	Maximum	0.799	0.755	0.650	0.648
Log Loss	Minimum	0.625	0.901	9.741	12.138
EOE	Minimum	0.730	0.758	1.022	1.032
Jaccard	Maximum	0.889	0.858	0.636	0.570
ROC AUC	Maximum	0.931	0.912	0.773	0.731
V Measure	Maximum	0.755	0.740	0.660	0.639
Rand	Maximum	0.919	0.899	0.820	0.795
Normalized Mutual Information	Maximum	0.755	0.740	0.660	0.639
Mutual Information	Maximum	1.213	1.181	0.969	0.887
Homogeneity	Maximum	0.754	0.734	0.602	0.551
Adjusted Rand	Maximum	0.749	0.691	0.524	0.497
Adjusted Mutual Info	Maximum	0.755	0.740	0.660	0.639

**Table 5:** Clusters using first synthetic dataset.

Method	FSBAGGMM
BIC	7
AIC	7
DI	4
MML	5
EoE	5
GT	5

**Table 6:** Clusters using second synthetic dataset.

Method	BAGGMM + FW
BIC	6
AIC	6
DI	6
MML	8
EoE	8
GT	8

**Table 7:** Count of observations generated for the second synthetic dataset.

Gaussian White Noise Parameters	Profile 1	Profile 2	Profile 3	Profile 4	Profile 5	Profile 6	Profile 7	Profile 8
μ = 0.001; σ = 0.2	445	448	450	444	449	447	442	455
μ = 0.01; σ = 0.2	442	449	448	448	448	452	445	448
μ = 0.1; σ = 0.2	442	452	455	449	447	443	447	445
μ = 0.05; σ = 0.3	445	448	444	451	453	447	442	450
μ = 0.01; σ = 0.3	460	459	458	468	457	455	466	457

**Table 8:** Mixture models’ clustering performance evaluation using the second synthetic dataset.

Performance Index (%)	FSBAGGMM	FSAGGMM	BAGGMM	AGGMM
ACC	91.856	88.746	88.481	87.769
TPR/Recall	67.459	54.969	53.862	51.021
PPV/Precision	66.482	55.753	56.402	54.291
MCC	63.813	50.402	49.908	46.726
F1-Score	67.422	54.983	53.922	51.078
TNR	95.347	93.570	93.418	93.012
NPV	95.456	93.921	93.926	93.528
FPR	4.653	6.430	6.582	6.988
FNR	32.541	45.031	46.138	48.979
FDR	33.518	44.247	43.598	45.709

**Table 9:** Mixture models’ clustering performance evaluation using the second synthetic dataset.

Performance Index	Optimal Performance Indicator	FSBAGGMM	FSAGGMM	BAGGMM	AGGMM
GOF	Minimum	22,539.820	36,474.842	50,310.225	48,011.423
CH	Maximum	2100.955	1766.797	1713.450	1674.616
S	Maximum	0.054	0.001	−0.052	−0.062
DB	Minimum	3.563	4.975	6.767	6.738
DI	Maximum	0.210	0.213	0.208	0.194
Xie and Benie	Minimum	2.883	3.619	3.683	3.784
Fowlkes Mallows	Maximum	0.574	0.486	0.518	0.503
Log Loss	Minimum	3.293	10.287	12.618	13.228
EOE	Minimum	0.620	0.637	0.685	0.675
Jaccard	Maximum	0.674	0.550	0.539	0.511
ROC AUC	Maximum	0.814	0.743	0.737	0.720
V Measure	Maximum	0.644	0.565	0.593	0.586
Rand	Maximum	0.881	0.836	0.831	0.821
Normalized Mutual Information	Maximum	0.644	0.565	0.593	0.586
Mutual Info	Maximum	1.303	1.088	1.114	1.093
Homogeneity	Maximum	0.627	0.523	0.536	0.526
Adjusted Rand	Maximum	0.502	0.384	0.407	0.385
Adjusted Mutual Info	Maximum	0.644	0.565	0.593	0.585

**Table 10:** Identified optimal number of clusters for the real-life smart meter dataset.

Model Selection Method	FSBAGGMM
BIC	3
AIC	3
DI	2
MML	3
EoE	4

**Table 11:** Mixture models’ clustering performance using the real-life smart meter dataset.

Performance Index	Metric’s Optimal Value	FSBAGGMM	FSAGGMM	BAGGMM	AGGMM
S	Maximum	0.250	0.216	0.228	0.176
CH	Maximum	7.377	5.824	6.671	5.594
DB	Minimum	16.951	23.832	20.626	24.577
DI	Maximum	0.253	0.238	0.249	0.224
Xie and Benie	Minimum	60.821	72.969	62.157	73.319
EOE	Minimum	1.460	1.764	1.613	1.822

**Table 12:** Consumption profile statistics for the year 2010.

Consumption Profile Cluster	Average Consumption (kWh)	Annual Consumption Responsibility	Clusters’ Proportion
1	6536.770	18.650%	64.600%
2	16,117.190	45.980%	1.700%
3	12,394.570	35.360%	33.700%

**Table 13:** Identified optimal number of clusters for the second real-life smart meter dataset.

Model Selection Method	FSBAGGMM
BIC	4
AIC	4
DI	4
MML	4
EoE	2

**Table 14:** Mixture models’ clustering performance using the second real-life smart meter dataset.

Performance Index	Metric’s Optimal Value	FSBAGGMM	FSAGGMM	BAGGMM	AGGMM
S	Maximum	0.319	0.288	0.265	0.189
CH	Maximum	1984.843	1078.837	545.442	243.243
DB	Minimum	1.050	1.075	2.583	3.108
DI	Maximum	0.027	0.023	0.019	0.012
Xie and Benie	Minimum	0.550	0.719	0.939	1.283
EOE	Minimum	0.315	0.434	0.442	0.453

**Table 15:** The mean values of the first seven smart meter data features.

Consumption Profile	Overnight RAP	Breakfast RAP	Daytime RAP	Evening RAP	Mean STD	Seasonal Score	WD-WE Diff. Score
1	0.686	0.937	1.041	1.344	0.810	0.883	0.458
2	0.664	1.050	0.956	1.411	1.127	1.025	1.557
3	0.672	0.959	1.011	1.381	0.974	2.062	0.553
4	0.860	0.981	0.916	1.249	1.169	4.445	0.591

## Data Availability

The data presented in this study are available on request from the corresponding author. The data are not publicly available due to ethical, legal or privacy issues.

## Appendix A. Important Partial Derivatives

(A1)
$$\begin{array}{cc} \frac{\partial \ln\Psi (X_{id}|\theta_{kd})}{\partial \mu_{kd}} & =\left\{\begin{array}{cc} \frac{\partial \ln g_{1}\left(X_{id}|\theta_{kd}\right)}{\mu_{kd}} & x<\mu_{kd}\\ \frac{\partial \ln g_{2}\left(X_{id}|\theta_{kd}\right)}{\mu_{kd}} & x\geq \mu_{kd} \end{array}\right.=\left\{\begin{array}{cc} -A\left(\lambda_{kd}\right)\lambda_{kd}\frac{{\left(\mu_{kd}-X_{id}\right)}^{\lambda_{kd}-1}}{\sigma_{l_{kd}}^{\lambda_{kd}}} & x<\mu_{kd}\\ A\left(\lambda_{kd}\right)\lambda_{kd}\frac{{\left(X_{id}-\mu_{kd}\right)}^{\lambda_{kd}-1}}{\sigma_{r_{kd}}^{\lambda_{kd}}} & x\geq \mu_{kd} \end{array}\right. \end{array}$$

(A2)
$$\begin{array}{cc} \frac{\partial L(X,\Theta_{M},Z,\varphi )}{\partial \mu_{kd}}= & \sum_{i=1}^{N}\frac{\omega_{d}p\left(x_{id}|\theta_{kd}\right)}{\zeta_{ikd}}p\left(k|\vec{X_{i}},\Theta_{M}\right)\\ & \times \left\{\begin{array}{cc} \begin{array}{cc} \; & \frac{\partial \ln g_{1}\left(X_{id}|\theta_{kd}\right)}{\partial \mu_{kd}}+\\ \; & \frac{\int_{\partial k}g_{1}\left(X_{id}|\theta_{kd}\right)\frac{\partial \ln g_{1}\left(X_{id}|\theta_{kd}\right)}{\partial \mu_{kd}}du}{\int_{\partial k}g_{1}\left(X_{id}|\theta_{kd}\right)du} \end{array} & x<\mu_{kd}\\ \begin{array}{cc} \; & \frac{\partial \ln g_{2}\left(X_{id}|\theta_{kd}\right)}{\partial \mu_{kd}}+\\ \; & \frac{\int_{\partial k}g_{2}\left(X_{id}|\theta_{kd}\right)\frac{\partial \ln g_{2}\left(X_{id}|\theta_{kd}\right)}{\partial \mu_{kd}}du}{\int_{\partial k}g_{2}\left(X_{id}|\theta_{kd}\right)du} \end{array} & x\geq \mu_{kd} \end{array}\right. \end{array}$$

(A3)
$$\begin{array}{cc} \frac{\partial \ln\Psi (X_{id}|\theta_{kd})}{\partial \sigma_{l_{kd}}} & =\left\{\begin{array}{cc} \frac{\partial \ln g_{1}\left(X_{id}|\theta_{kd}\right)}{\partial \sigma l_{kd}} & x<\mu_{kd}\\ \frac{\partial \ln g_{2}\left(X_{id}|\theta_{kd}\right)}{\partial \sigma l_{kd}} & x\geq \mu_{kd} \end{array}\right.\\ & =\left\{\begin{array}{cc} A\left(\lambda_{kd}\right)\lambda_{kd}\frac{{\left(\mu_{kd}-X_{id}\right)}^{\lambda_{kd}}}{\sigma_{l_{kd}}^{\lambda_{kd}+1}}-\frac{1}{\sigma_{l_{kd}}+\sigma_{r_{kd}}} & x<\mu_{kd}\\ -\frac{1}{\sigma_{l_{kd}}+\sigma_{r_{kd}}} & x\geq \mu_{kd} \end{array}\right. \end{array}$$

(A4)
$$\begin{array}{cc} \frac{\partial L(X,\Theta_{M},Z,\varphi )}{\partial \sigma_{l_{kd}}}= & \sum_{i=1}^{N}\frac{\omega_{d}p\left(x_{id}|\theta_{kd}\right)}{\zeta_{ikd}}p\left(k|\vec{X_{i}},\Theta_{M}\right)\\ & \times \left\{\begin{array}{cc} \begin{array}{c} \frac{\partial \ln g_{1}\left(X_{id}|\theta_{kd}\right)}{\partial \sigma l_{kd}}+\frac{\int_{\partial k}g_{1}\left(X_{id}|\theta_{kd}\right)\frac{\partial \ln g_{1}\left(X_{id}|\theta_{kd}\right)}{\partial \sigma l_{kd}}du}{\int_{\partial k}g_{1}\left(X_{id}|\theta_{kd}\right)du} \end{array} & x<\mu_{kd}\\ \begin{array}{c} \frac{\partial \ln g_{2}\left(X_{id}|\theta_{kd}\right)}{\partial \sigma l_{kd}}+\frac{\int_{\partial k}g_{2}\left(X_{id}|\theta_{kd}\right)\frac{\partial \ln g_{2}\left(X_{id}|\theta_{kd}\right)}{\partial \sigma l_{kd}}du}{\int_{\partial k}g_{2}\left(X_{id}|\theta_{kd}\right)du} \end{array} & x\geq \mu_{kd} \end{array}\right. \end{array}$$

(A5)
$$\begin{array}{cc} \frac{\partial \ln\Psi (X_{id}|\theta_{kd})}{\partial \sigma_{r_{kd}}} & =\left\{\begin{array}{cc} \frac{\partial \ln g_{1}\left(X_{id}|\theta_{kd}\right)}{\partial \sigma_{r_{kd}}} & x<\mu_{kd}\\ \frac{\partial \ln g_{2}\left(X_{id}|\theta_{kd}\right)}{\partial \sigma_{r_{kd}}} & x\geq \mu_{kd} \end{array}\right.=\left\{\begin{array}{cc} -\frac{1}{\sigma_{l_{kd}}+\sigma_{r_{kd}}} & x<\mu_{kd}\\ \frac{A\left(\lambda_{kd}\right)\lambda_{kd}}{\sigma_{r_{kd}}}\frac{{\left(X_{id}-\mu_{kd}\right)}^{\lambda_{kd}}}{\sigma_{r_{kd}}^{\lambda_{kd}}}-\frac{1}{\sigma_{l_{kd}}+\sigma_{r_{kd}}} & x\geq \mu_{kd} \end{array}\right. \end{array}$$

(A6)
$$\begin{array}{cc} \frac{\partial L(X,\Theta_{M},Z,\varphi )}{\partial \sigma_{r_{kd}}}= & \sum_{i=1}^{N}\frac{\omega_{d}p\left(x_{id}|\theta_{kd}\right)}{\zeta_{ikd}}p\left(k|\vec{X_{i}},\Theta_{M}\right)\\ & \times \left\{\begin{array}{cc} \begin{array}{c} \frac{\partial \ln g_{1}\left(X_{id}|\theta_{kd}\right)}{\partial \sigma r_{kd}}+\frac{\int_{\partial k}g_{1}\left(X_{id}|\theta_{kd}\right)\frac{\partial \ln g_{1}\left(X_{id}|\theta_{kd}\right)}{\partial \sigma r_{kd}}du}{\int_{\partial k}g_{1}\left(X_{id}|\theta_{kd}\right)du} \end{array} & x<\mu_{kd}\\ \begin{array}{c} \frac{\partial \ln g_{2}\left(X_{id}|\theta_{kd}\right)}{\partial \sigma r_{kd}}+\frac{\int_{\partial k}g_{2}\left(X_{id}|\theta_{kd}\right)\frac{\partial \ln g_{2}\left(X_{id}|\theta_{kd}\right)}{\partial \sigma r_{kd}}du}{\int_{\partial k}g_{2}\left(X_{id}|\theta_{kd}\right)du} \end{array} & x\geq \mu_{kd} \end{array}\right. \end{array}$$

(A7)
$$\begin{array}{cc} \frac{\partial \ln\Psi (X_{id}|\theta_{kd})}{\partial \lambda_{kd}} & =\left\{\begin{array}{cc} \frac{\partial \ln g_{1}\left(X_{id}|\theta_{kd}\right)}{\partial \lambda_{kd}} & x<\mu_{kd}\\ \frac{\partial \ln g_{2}\left(X_{id}|\theta_{kd}\right)}{\partial \lambda_{kd}} & x\geq \mu_{kd} \end{array}\right.\\ \; & =\left\{\begin{array}{cc} \begin{array}{cc} \frac{1}{\lambda_{kd}} & +\frac{3(\psi \left(1/\lambda_{kd}\right)-\psi \left(3/\lambda_{kd}\right))}{2\lambda_{kd}^{2}}-{\left(\frac{\mu_{kd}-X_{id}}{\sigma_{l_{kd}}}\right)}^{\lambda_{kd}}A\left(\lambda_{kd}\right)\\ \; & \times [\frac{1}{2}\ln\left(\frac{\Gamma (3/\lambda_{kd})}{\Gamma (1/\lambda_{kd})}\right)\\ & +\frac{\left(\psi \left(1/\lambda_{kd}\right)-3\psi \left(3/\lambda_{kd}\right)\right)}{2\lambda_{kd}}+\ln\left(\frac{\mu_{kd}-X_{id}}{\sigma_{l_{kd}}}\right)] \end{array} & x<\mu_{kd}\\ \begin{array}{cc} \frac{1}{\lambda_{kd}} & +\frac{3(\psi \left(1/\lambda_{kd}\right)-\psi \left(3/\lambda_{kd}\right))}{2\lambda_{kd}^{2}}-{\left(\frac{X_{id}-\mu_{kd}}{\sigma_{r_{kd}}}\right)}^{\lambda_{kd}}A\left(\lambda_{kd}\right)\\ \; & \times [\frac{1}{2}\ln\left(\frac{\Gamma (3/\lambda_{kd})}{\Gamma (1/\lambda_{kd})}\right)\\ & +\frac{\left(\psi \left(1/\lambda_{kd}\right)-3\psi \left(3/\lambda_{kd}\right)\right)}{2\lambda_{kd}}+\ln\left(\frac{X_{id}-\mu_{kd}}{\sigma_{r_{kd}}}\right)] \end{array} & x\geq \mu_{kd} \end{array}\right. \end{array}$$

(A8)
$$\begin{array}{cc} \frac{\partial L(X,\Theta_{M},Z,\varphi )}{\partial \lambda_{kd}}= & \sum_{i=1}^{N}\frac{\omega_{d}p\left(x_{id}|\theta_{kd}\right)}{\zeta_{ikd}}p\left(k|\vec{X_{i}},\Theta_{M}\right)\\ \; & \times \left\{\begin{array}{cc} \begin{array}{c} \frac{\partial \ln g_{1}\left(X_{id}|\theta_{kd}\right)}{\partial \lambda_{kd}}+\frac{\int_{\partial k}g_{1}\left(X_{id}|\theta_{kd}\right)\frac{\partial \ln g_{1}\left(X_{id}|\theta_{kd}\right)}{\partial \lambda_{kd}}du}{\int_{\partial k}g_{1}\left(X_{id}|\theta_{kd}\right)du} \end{array} & x<\mu_{kd}\\ \begin{array}{c} \frac{\partial \ln g_{2}\left(X_{id}|\theta_{kd}\right)}{\partial \lambda_{kd}}+\frac{\int_{\partial k}g_{2}\left(X_{id}|\theta_{kd}\right)\frac{\partial \ln g_{2}\left(X_{id}|\theta_{kd}\right)}{\partial \lambda_{kd}}du}{\int_{\partial k}g_{2}\left(X_{id}|\theta_{kd}\right)du} \end{array} & x\geq \mu_{kd} \end{array}\right. \end{array}$$

(A9)
$$\begin{array}{cc} \; & \frac{\partial^{2}L\left(X,\Theta_{M},Z,\varphi \right)}{\partial \mu_{kd}^{2}}=\sum_{i=1}^{N}\frac{\omega_{d}p\left(x_{id}|\theta_{kd}\right)}{\zeta_{ikd}}p\left(k|\vec{X_{i}},\Theta_{M}\right)\\ \; & \times \left\{\begin{array}{cc} \begin{array}{cc} \; & \frac{\partial^{2}\ln g_{1}\left(X_{id}|\theta_{kd}\right)}{\partial \mu_{kd}^{2}}\\ & +\frac{\int_{\partial k}g_{1}\left(X_{id}|\theta_{kd}\right)\left[{\left(\frac{\partial \ln g_{1}\left(X_{id}|\theta_{kd}\right)}{\partial \mu_{kd}}\right)}^{2}+\frac{\partial^{2}\ln g_{1}\left(X_{id}|\theta_{kd}\right)}{\partial \mu_{kd}^{2}}\right]du}{\int_{\partial k}g_{1}\left(X_{id}|\theta_{kd}\right)du}\\ \; & -\frac{{\left(\int_{\partial k}g_{1}\left(X_{id}|\theta_{kd}\right)\frac{\partial \ln g_{1}\left(X_{id}|\theta_{kd}\right)}{\partial \mu_{kd}}du\right)}^{2}}{{\left(\int_{\partial k}g_{1}\left(X_{id}|\theta_{kd}\right)du\right)}^{2}} \end{array} & x<\mu_{kd}\\ \begin{array}{cc} \; & \frac{\partial^{2}\ln g_{2}\left(X_{id}|\theta_{kd}\right)}{\partial \mu_{kd}^{2}}+\\ & \frac{\int_{\partial k}g_{2}\left(X_{id}|\theta_{kd}\right)\left[{\left(\frac{\partial \ln g_{2}\left(X_{id}|\theta_{kd}\right)}{\partial \mu_{kd}}\right)}^{2}+\frac{\partial^{2}\ln g_{2}\left(X_{id}|\theta_{kd}\right)}{\partial \mu_{kd}^{2}}\right]du}{\int_{\partial k}g_{2}\left(X_{id}|\theta_{kd}\right)du}\\ \; & -\frac{{\left(\int_{\partial k}g_{2}\left(X_{id}|\theta_{kd}\right)\frac{\partial \ln g_{2}\left(X_{id}|\theta_{kd}\right)}{\partial \mu_{kd}}du\right)}^{2}}{{\left(\int_{\partial k}g_{2}\left(X_{id}|\theta_{kd}\right)du\right)}^{2}} \end{array} & x\geq \mu_{kd} \end{array}\right. \end{array}$$

(A10)
$$\begin{array}{cc} \frac{\partial^{2}\ln\Psi \left(X_{id}|\theta_{kd}\right)}{\partial \mu_{kd}^{2}} & =\left\{\begin{array}{cc} \frac{\partial^{2}\ln g_{1}\left(X_{id}|\theta_{kd}\right)}{\partial \mu_{kd}^{2}} & x<\mu_{kd}\\ \frac{\partial^{2}\ln g_{2}\left(X_{id}|\theta_{kd}\right)}{\partial \mu_{kd}^{2}} & x\geq \mu_{kd} \end{array}\right.\\ & =\left\{\begin{array}{cc} -A\left(\lambda_{kd}\right)\lambda_{kd}\left(\lambda_{kd}-1\right)\frac{{\left(\partial \mu_{kd}-X_{id}\right)}^{\lambda_{kd}-2}}{\sigma_{kd}^{\lambda_{kd}}} & x<\mu_{kd}\\ -A\left(\lambda_{kd}\right)\lambda_{kd}\left(\lambda_{kd}-1\right)\frac{{\left(X_{id}-\mu_{kd}\right)}^{\lambda_{kd}-2}}{\sigma_{kd}^{\lambda_{kd}}} & x\geq \mu_{kd} \end{array}\right. \end{array}$$

(A11)
$$\begin{array}{cc} \; & \frac{\partial^{2}L\left(X,\Theta_{M},Z,\varphi \right)}{\partial \sigma_{l_{kd}}^{2}}=\sum_{i=1}^{N}\frac{\omega_{d}p\left(x_{id}|\theta_{kd}\right)}{\zeta_{ikd}}p\left(k|\vec{X_{i}},\Theta_{M}\right)\\ \; & \times \left\{\begin{array}{cc} \begin{array}{cc} \; & \frac{\partial^{2}\ln g_{1}\left(X_{id}|\theta_{kd}\right)}{\partial \sigma_{l_{kd}}^{2}}+\frac{\int_{\partial k}g_{1}\left(X_{id}|\theta_{kd}\right)\left[{\left(\frac{\partial \ln g_{1}\left(X_{id}|\theta_{kd}\right)}{\partial \sigma_{l_{kd}}}\right)}^{2}+\frac{\partial^{2}\ln g_{1}\left(X_{id}|\theta_{kd}\right)}{\partial \sigma_{l_{kd}}^{2}}\right]du}{\int_{\partial k}g_{1}\left(X_{id}|\theta_{kd}\right)du}\\ \; & -\frac{{\left(\int_{\partial k}g_{1}\left(X_{id}|\theta_{kd}\right)\frac{\partial \ln g_{1}\left(X_{id}|\theta_{kd}\right)}{\partial \sigma_{l_{kd}}}du\right)}^{2}}{{\left(\int_{\partial k}g_{1}\left(X_{id}|\theta_{kd}\right)du\right)}^{2}} \end{array} & x<\mu_{kd}\\ \begin{array}{cc} \; & \frac{\partial^{2}\ln g_{2}\left(X_{id}|\theta_{kd}\right)}{\partial \sigma_{l_{kd}}^{2}}+\frac{\int_{\partial k}g_{2}\left(X_{id}|\theta_{kd}\right)\left[{\left(\frac{\partial \ln g_{2}\left(X_{id}|\theta_{kd}\right)}{\partial \sigma_{l_{kd}}}\right)}^{2}+\frac{\partial^{2}\ln g_{2}\left(X_{id}|\theta_{kd}\right)}{\partial \sigma_{l_{kd}}^{2}}\right]du}{\int_{\partial k}g_{2}\left(X_{id}|\theta_{kd}\right)du}\\ \; & -\frac{{\left(\int_{\partial k}g_{2}\left(X_{id}|\theta_{kd}\right)\frac{\partial \ln g_{2}\left(X_{id}|\theta_{kd}\right)}{\partial \sigma_{l_{kd}}}du\right)}^{2}}{{\left(\int_{\partial k}g_{2}\left(X_{id}|\theta_{kd}\right)du\right)}^{2}} \end{array} & x\geq \mu_{kd} \end{array}\right. \end{array}$$

(A12)
$$\begin{array}{cc} \frac{\partial^{2}\ln\Psi \left(X_{id}|\theta_{kd}\right)}{\partial \sigma_{l_{kd}}^{2}} & =\left\{\begin{array}{cc} \frac{\partial^{2}\ln g_{1}\left(X_{id}|\theta_{kd}\right)}{\partial \sigma_{l_{kd}}^{2}} & x<\mu_{kd}\\ \frac{\partial^{2}\ln g_{2}\left(X_{id}|\theta_{kd}\right)}{\partial \sigma_{l_{kd}}^{2}} & x\geq \mu_{kd} \end{array}\right.\\ & =\left\{\begin{array}{cc} -\left(\lambda_{kd}+1\right)A\left(\lambda_{kd}\right)\lambda_{kd}\frac{{\left(\mu_{kd}-X_{id}\right)}^{\lambda_{kd}}}{\sigma_{l_{kd}}^{\lambda_{kd}+2}}+\frac{1}{{\left(\sigma_{l_{kd}}+\sigma_{r_{kd}}\right)}^{2}} & x<\mu_{kd}\\ \frac{1}{{\left(\sigma_{l_{kd}}+\sigma_{r_{kd}}\right)}^{2}} & x\geq \mu_{kd} \end{array}\right. \end{array}$$

(A13)
$$\begin{array}{cc} \; & \frac{\partial^{2}L\left(X,\Theta_{M},Z,\varphi \right)}{\partial \sigma_{r_{kd}}^{2}}=\sum_{i=1}^{N}\frac{\omega_{d}p\left(x_{id}|\theta_{kd}\right)}{\zeta_{ikd}}p\left(k|\vec{X_{i}},\Theta_{M}\right)\\ \; & \times \left\{\begin{array}{cc} \begin{array}{cc} \; & \frac{\partial^{2}\ln g_{1}\left(X_{id}|\theta_{kd}\right)}{\partial \sigma_{r_{kd}}^{2}}+\frac{\int_{\partial k}g_{1}\left(X_{id}|\theta_{kd}\right)\left[{\left(\frac{\partial \ln g_{1}\left(X_{id}|\theta_{kd}\right)}{\partial \sigma_{r_{kd}}}\right)}^{2}+\frac{\partial^{2}\ln g_{1}\left(X_{id}|\theta_{kd}\right)}{\partial \sigma_{r_{kd}}^{2}}\right]du}{\int_{\partial k}g_{1}\left(X_{id}|\theta_{kd}\right)du}\\ \; & -\frac{{\left(\int_{\partial k}g_{1}\left(X_{id}|\theta_{kd}\right)\frac{\partial \ln g_{1}\left(X_{id}|\theta_{kd}\right)}{\partial \sigma_{r_{kd}}}du\right)}^{2}}{{\left(\int_{\partial k}g_{1}\left(X_{id}|\theta_{kd}\right)du\right)}^{2}} \end{array} & x<\mu_{kd}\\ \begin{array}{cc} \; & \frac{\partial^{2}\ln g_{2}\left(X_{id}|\theta_{kd}\right)}{\partial \sigma_{r_{kd}}^{2}}+\frac{\int_{\partial k}g_{2}\left(X_{id}|\theta_{kd}\right)\left[{\left(\frac{\partial \ln g_{2}\left(X_{id}|\theta_{kd}\right)}{\partial \sigma_{r_{kd}}}\right)}^{2}+\frac{\partial^{2}\ln g_{2}\left(X_{id}|\theta_{kd}\right)}{\partial \sigma_{r_{kd}}^{2}}\right]du}{\int_{\partial k}g_{2}\left(X_{id}|\theta_{kd}\right)du}\\ \; & -\frac{{\left(\int_{\partial k}g_{2}\left(X_{id}|\theta_{kd}\right)\frac{\partial \ln g_{2}\left(X_{id}|\theta_{kd}\right)}{\partial \sigma_{r_{kd}}}du\right)}^{2}}{{\left(\int_{\partial k}g_{2}\left(X_{id}|\theta_{kd}\right)du\right)}^{2}} \end{array} & x\geq \mu_{kd} \end{array}\right. \end{array}$$

(A14)
$$\begin{array}{cc} \frac{\partial^{2}\ln\Psi \left(X_{id}|\theta_{kd}\right)}{\partial \sigma {r_{kd}}^{2}} & =\left\{\begin{array}{cc} \frac{\partial^{2}\ln g_{1}\left(X_{id}|\theta_{kd}\right)}{\partial \sigma_{r_{kd}}^{2}} & x<\mu_{kd}\\ \frac{\partial^{2}\ln g_{2}\left(X_{id}|\theta_{kd}\right)}{\partial \sigma_{r_{kd}}^{2}} & x\geq \mu_{kd} \end{array}\right.\\ & =\left\{\begin{array}{cc} \frac{1}{{\left(\sigma_{l_{kd}}+\sigma_{r_{kd}}\right)}^{2}} & x<\mu_{kd}\\ -\left(\lambda_{kd}+1\right)A\left(\lambda_{kd}\right)\lambda_{kd}\frac{{\left(X_{id}-\mu_{kd}\right)}^{\lambda_{kd}}}{\sigma_{r_{kd}}^{\lambda_{kd}+2}}+\frac{1}{{\left(\sigma_{l_{kd}}+\sigma_{r_{kd}}\right)}^{2}} & x\geq \mu_{kd} \end{array}\right. \end{array}$$

(A15)
$$\begin{array}{cc} \; & \frac{\partial^{2}L\left(X,\Theta_{M},Z,\varphi \right)}{\partial \lambda_{kd}^{2}}=\sum_{i=1}^{N}\frac{\omega_{d}p\left(x_{id}|\theta_{kd}\right)}{\zeta_{ikd}}p\left(k|\vec{X_{i}},\Theta_{M}\right)\\ \; & \times \left\{\begin{array}{cc} \begin{array}{cc} \; & \frac{\partial^{2}\ln g_{1}\left(X_{id}|\theta_{kd}\right)}{\partial \lambda_{kd}^{2}}+\frac{\int_{\partial k}g_{1}\left(X_{id}|\theta_{kd}\right)\left[{\left(\frac{\partial \ln g_{1}\left(X_{id}|\theta_{kd}\right)}{\partial \lambda_{kd}}\right)}^{2}+\frac{\partial^{2}\ln g_{1}\left(X_{id}|\theta_{kd}\right)}{\partial \lambda_{kd}^{2}}\right]du}{\int_{\partial k}g_{1}\left(X_{id}|\theta_{kd}\right)du}\\ \; & -\frac{{\left(\int_{\partial k}g_{1}\left(X_{id}|\theta_{kd}\right)\frac{\partial \ln g_{1}\left(X_{id}|\theta_{kd}\right)}{\partial \lambda_{kd}}du\right)}^{2}}{{\left(\int_{\partial k}g_{1}\left(X_{id}|\theta_{kd}\right)du\right)}^{2}} \end{array} & x<\mu_{kd}\\ \begin{array}{cc} \; & \frac{\partial^{2}\ln g_{2}\left(X_{id}|\theta_{kd}\right)}{\partial \lambda_{kd}^{2}}+\frac{\int_{\partial k}g_{2}\left(X_{id}|\theta_{kd}\right)\left[{\left(\frac{\partial \ln g_{2}\left(X_{id}|\theta_{kd}\right)}{\partial \lambda_{kd}}\right)}^{2}+\frac{\partial^{2}\ln g_{2}\left(X_{id}|\theta_{kd}\right)}{\partial \lambda_{kd}^{2}}\right]du}{\int_{\partial k}g_{2}\left(X_{id}|\theta_{kd}\right)du}\\ \; & -\frac{{\left(\int_{\partial k}g_{2}\left(X_{id}|\theta_{kd}\right)\frac{\partial \ln g_{2}\left(X_{id}|\theta_{kd}\right)}{\partial \lambda_{kd}}du\right)}^{2}}{{\left(\int_{\partial k}g_{2}\left(X_{id}|\theta_{kd}\right)du\right)}^{2}} \end{array} & x\geq \mu_{kd} \end{array}\right. \end{array}$$

(A16)
$$\begin{array}{cc} & \frac{\partial^{2}\ln\Psi \left(X_{id}|\theta_{kd}\right)}{\partial \lambda_{kd}^{2}}=\left\{\begin{array}{cc} \frac{\partial^{2}\ln g_{1}\left(X_{id}|\theta_{kd}\right)}{\partial \lambda_{kd}^{2}} & x<\mu_{kd}\\ \frac{\partial^{2}\ln g_{2}\left(X_{id}|\theta_{kd}\right)}{\partial \lambda_{kd}^{2}} & x\geq \mu_{kd} \end{array}\right.\\ & =\left\{\begin{array}{cc} \begin{array}{cc} -\frac{1}{\lambda_{kd}^{2}} & +\frac{3(\psi^{\prime }\left(3/\lambda_{kd}\right)-\psi^{\prime }\left(1/\lambda_{kd}\right))}{2\lambda_{kd}^{4}}-\frac{3(\psi \left(1/\lambda_{kd}\right)-\psi \left(3/\lambda_{kd}\right))}{\lambda_{kd}^{3}}\\ & -{\left(\frac{\mu_{kd}-X_{id}}{\sigma_{l_{kd}}}\right)}^{\lambda_{kd}}A\left(\lambda_{kd}\right)\\ & \times [\frac{\left(9\psi^{\prime }\left(3/\lambda_{kd}\right)-\psi^{\prime }\left(1/\lambda_{kd}\right)\right)}{2\lambda_{kd}^{3}}\\ & +[\frac{1}{2}\ln\left(\frac{\Gamma (3/\lambda_{kd})}{\Gamma (1/\lambda_{kd})}\right)+\frac{\left(\psi \left(1/\lambda_{kd}\right)-3\psi \left(3/\lambda_{kd}\right)\right)}{2\lambda_{kd}}\\ & +\ln\left(\frac{\mu_{kd}-X_{id}}{\sigma_{l_{kd}}}\right){]}^{2}] \end{array} & x<\mu_{kd}\\ \begin{array}{cc} -\frac{1}{\lambda_{kd}^{2}} & +\frac{3(\psi^{\prime }\left(3/\lambda_{kd}\right)-\psi^{\prime }\left(1/\lambda_{kd}\right))}{2\lambda_{kd}^{4}}-\frac{3(\psi \left(1/\lambda_{kd}\right)-\psi \left(3/\lambda_{kd}\right))}{\lambda_{kd}^{3}}\\ & -{\left(\frac{X_{id}-\mu_{kd}}{\sigma_{r_{kd}}}\right)}^{\lambda_{kd}}A\left(\lambda_{kd}\right)\\ & \times [\frac{\left(9\psi^{\prime }\left(3/\lambda_{kd}\right)-\psi^{\prime }\left(1/\lambda_{kd}\right)\right)}{2\lambda_{kd}^{3}}\\ & +[\frac{1}{2}\ln\left(\frac{\Gamma (3/\lambda_{kd})}{\Gamma (1/\lambda_{kd})}\right)+\frac{\left(\psi \left(1/\lambda_{kd}\right)-3\psi \left(3/\lambda_{kd}\right)\right)}{2\lambda_{kd}}\\ & +\ln\left(\frac{X_{id}-\mu_{kd}}{\sigma_{r_{kd}}}\right){]}^{2}] \end{array} & x\geq \mu_{kd} \end{array}\right. \end{array}$$
